# Multi-modal MRI for objective diagnosis and outcome prediction in depression

**DOI:** 10.1016/j.nicl.2024.103682

**Published:** 2024-10-10

**Authors:** Jesper Pilmeyer, Rolf Lamerichs, Sjir Schielen, Faroeq Ramsaransing, Vivianne van Kranen-Mastenbroek, Jacobus F.A. Jansen, Marcel Breeuwer, Svitlana Zinger

**Affiliations:** a Department of Electrical Engineering, Eindhoven University of Technology, Groene Loper 19, 5612 AE Eindhoven, the Netherlands; b Department of Research and Development, Epilepsy Centre Kempenhaeghe, Sterkselseweg 65, 5590 AB Heeze, the Netherlands; c Department of Medical Image Acquisitions, Philips Research, High Tech Campus 34, 5656 AE Eindhoven, the Netherlands; d Department of Psychiatry, Amsterdam University Medical Center, Meibergdreef 5, 1105 AZ Amsterdam, the Netherlands; e Mental Health and Neuroscience Research Institute, Maastricht University, Minderbroedersberg 4-6, 6211 LK Maastricht, the Netherlands; f Academic Center for Epileptology, Kempenhaeghe and Maastricht University Medical Centre, Heeze and Maastricht, the Netherlands; g Department of Clinical Neurophysiology, Maastricht University Medical Centre, P. Debyelaan 25, 6229 HX Maastricht, the Netherlands; h Department of Radiology and Nuclear Medicine, Maastricht University Medical Centre, P. Debyelaan 25, 6229 HX Maastricht, the Netherlands; i Department of Biomedical Engineering, Eindhoven University of Technology, Groene Loper 5, 5612 AE Eindhoven, the Netherlands

**Keywords:** Major depressive disorder_1_, Prognosis_2_, Multi-modal_3_, MRI_4_, Machine learning_5_

## Abstract

**Research Purpose:**

The low treatment effectiveness in major depressive disorder (MDD) may be caused by the subjectiveness in clinical examination and the lack of quantitative tests. Objective biomarkers derived from magnetic resonance imaging (MRI) may support clinical experts during decision-making. Numerous studies have attempted to identify such MRI-based biomarkers. However, the majority is uni-modal (based on a single MRI modality) and focus on either MDD diagnosis or outcome. Uncertainty remains regarding whether key features or classification models for diagnosis may also be used for outcome prediction. Therefore, we aim to find multi-modal predictors of both, MDD diagnosis and outcome. By addressing these research questions using the same dataset, we eliminate between-study confounding factors.

Various structural (T_1_-weighted, T_2_-weighted, diffusion tensor imaging (DTI)) and functional (resting-state and task-based functional MRI) scans were acquired from 32 MDD and 31 healthy control (HC) subjects during the first visit at the investigational site (baseline). Depression severity was assessed at baseline and 6 months later. Features were extracted from the baseline MRI images with different modalities. Binary 6-months negative and positive outcome (NO; PO) classes were defined based on relative (to baseline) change in depression severity. Support vector machine models were employed to separate MDD from HC (diagnosis) and NO from PO subjects (outcome). Classification was performed through a uni-modal (features from a single MRI modality) and multi-modal (combination of features from different modalities) approach.

**Principal Results:**

Our results show that DTI features yielded the highest uni-modal performance for diagnosis and outcome prediction: mean diffusivity (AUC (area under the curve) = 0.701) and the sum of streamline weights (AUC = 0.860), respectively. Multi-modal ensemble classifiers with T_1_-weighted, resting-state functional MRI and DTI features improved classification performance for both diagnosis and outcome (AUC = 0.746 and 0.932, respectively). Feature analyses revealed that the most important features were located in frontal, limbic and parietal areas. However, the modality or location of these features was different between diagnostic and prognostic models.

**Major Conclusions:**

Our findings suggest that combining features from different MRI modalities predict MDD diagnosis and outcome with higher performance. Furthermore, we demonstrated that the most important features for MDD diagnosis were different and located in other brain regions than those for outcome. This longitudinal study contributes to the identification of objective biomarkers of MDD and its outcome. Follow-up studies may further evaluate the generalizability of our models in larger or multi-center cohorts.

## Highlights

• Multi-modal MRI improved diagnosis and outcome prediction compared to single-modal.
• Key predictors were in frontal, limbic, parietal regions but varied between models.
• Multi-modal MRI may explain MDD’s pathophysiology and heterogeneity more accurately.

## 1 Introduction

Approximately one out of six people will experience a depressive episode during their lifetime (Malhi and Mann, 2018). Despite a high impact on daily functioning and quality of life, treatment effectiveness of major depressive disorder is still relatively low, with no less than two-thirds of the patients remaining symptomatic after first-line treatment and about a third not achieving remission after finishing four subsequent treatments (Holtzheimer and Mayberg, 2011, Malhi and Mann, 2018). One of the underlying causes for this may be the subjective decision-making by clinical experts.

In order to support these critical judgements with more objective measures, numerous researchers have attempted to discover biomarkers for diagnosis, prognosis and treatment navigation purposes (Abi-Dargham and Horga, 2016, Hacimusalar and Eşel, 2018, Kang and Cho, 2020). A common approach is through magnetic resonance imaging (MRI), which can provide structural and functional insights into the brain. Common MRI modalities include T_1_-weighted, T_2_-weighted, diffusion tensor imaging (DTI) and functional MRI (fMRI). Findings have linked structural (Abe et al., 2010, Atkinson et al., 2014, Brown et al., 2019, Cole et al., 2011, Grieve et al., 2016, Hamilton et al., 2008, Korgaonkar et al., 2014, Kraus et al., 2019, Samuels et al., 2015) and functional (Dai et al., 2019, Dichter et al., 2015, Lener and Iosifescu, 2015, Perlman et al., 2019, Stuhrmann et al., 2011) abnormalities to MDD and its treatment outcome. However, consistent depression biomarkers remain elusive (Atkinson et al., 2014, McKinnon et al., 2009) due to, amongst others, varying study designs, participant selection, and MRI protocols, as well as MDD symptom heterogeneity (Malhi and Mann, 2018).

Multi-modal MRI studies can mitigate part of these challenges by identification of biomarkers across different domains. Currently, multi-modal MRI research in MDD is scarce. Two recent studies achieved 73.5 % and 86.7 % accuracy in classifying MDD patients versus healthy controls (HCs) through combination of brain region volumes, cortical thickness, FC, and FA (Li et al., 2024, Zhang et al., 2023). Similar findings have been reported in studies focusing on prediction on remission and treatment outcome (Ma et al., 2023, Poirot et al., 2024). For example, a recent multi-site study including 229 MDD patients, predicted sertraline treatment response at 8 weeks with an AUC of 0.73 (Poirot et al., 2024). Crucially, integration of MRI measures derived from T_1_-weighted, resting-state fMRI, DTI and arterial spin labeling scans, demonstrated higher performance compared to a single modality.

A limitation of these studies is that they focused on either diagnosis or outcome prediction. Currently, it is unknown whether diagnostic findings can also be utilized for outcome prediction. It is crucial to investigate both research questions since future prognostic biomarker studies may benefit (or not) from formulating hypotheses, replicating models or defining regions-of-interest based on diagnostic findings and vice versa. However, comparison between studies is not straightforward due to differences in e.g. selection criteria and acquisition parameters. By conducting these experiments on a single dataset, are eliminated and findings can be compared more easily. The current multi-modal MRI study is the first, according to the authors’ knowledge, to objectively classify both, MDD and its outcome.

Additionally, most prognostic MDD studies compared between groups of patients receiving different treatments and control groups. These patients are often recruited in specialized mental health care, resulting in a sample that may not resemble the general MDD population as the depression severity and treatment use is relatively homogeneous (Schmaal et al., 2015). Therefore, we conducted our study in a naturalistic setting, including patients with varying depression severity, (mild to more chronic), depression history (no or multiple remissions) and treatment use. As such design captures more of the heterogeneity, findings and models may be more generalizable to the general MDD population.

In this study, MDD and HC participants receiving no, psychotherapy, and/or medication treatment were scanned at baseline. Symptom severity of the former group was re-assessed at 6-months follow-up. Features derived from T_1_- and T_2_-weighted, fMRI and diffusion MRI scans were fed to machine learning models. These included relatively novel features, such as amygdalar and hippocampal subregion volumes, functional measures reflecting different types of brain network interactions, and more biologically representative connectivity measures assessed along white matter tracts. Linear support vector machine (SVM) models were implemented to distinguish MDD from HC participants and MDD participants with a 6-months positive outcome (PO) from those with a negative outcome (NO), labeled by changes in symptom severity. Based on the state-of-the-art literature, We expected the highest diagnostic and prognostic performance from combining features across MRI modalities in the frontolimbic areas.

## 2 Material and methods

**Fig. 1:**
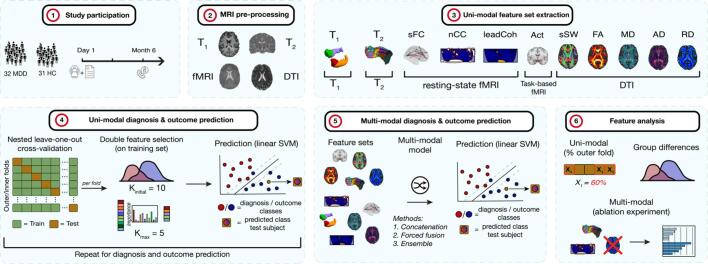
**Study workflow** 1) 32 major depressive disorder (MDD) and 31 healthy control (HC) subjects participated in the study. The depression severity of the MDD group was obtained at day 1 and after 6 months to define outcome groups. 2) Pre-processing of anatomical and functional scans was performed. 3) In total, 11 different feature sets were obtained: T_1_-weighted, T_2_-weighted, static functional connectivity (sFC), number of coherence clusters (nCC), lead coherence (leadCoh), activity (Act), sum of streamline weights (sSW), fractional anisotropy (FA), and mean/axial/radial diffusivity (MD/AD/RD). 4) For each set, linear support vector machine (SVM) classifiers were applied, combined with double feature selection in a nested leave-one-out cross-validation approach. 5) Feature sets were combined to test multi-modal classifiers using 3 different methods. 6) An elaborate feature analysis was conducted to assess the highest feature (element) contributions. Abbreviations: fMRI = functional magnetic resonance imaging; DTI = diffusion tensor imaging.

This section describes the conducted steps from participant inclusion until classification performance estimation for the uni- and multi-modal approaches. First, participant selection and inclusion results are displayed, followed by the estimation of clinical assessments to define the outcome group. Then, the MRI acquisition, pre-processing and feature extraction methods are explained. Finally, classification for the uni- and multi-modal details are provided, followed by a section describing the elaborate analyses to evaluate the most important features. A graphic overview can be found in Fig. 1.

### 2.1 Participants

A total of 63 subjects participated in the study: 32 adults with confirmed MDD diagnosis (age 43.8 ± 13.4 years, 12/20 male/females) and 31 age- and sex-matched healthy controls (age 40.4 ± 15.5 years, 11/20 male/females). For participant demographics and depression-related information, see Table 1 below. From the MDD group, 22 patients received one or two types of antidepressants at baseline (11 selective serotonin reuptake inhibitors, 7 serotonin and norepinephrine reuptake inhibitors, 3 noradrenaline and specific serotonergic antidepressants, 3 tricyclic antidepressants and 1 serotonin antagonist and reuptake inhibitors). A total of 24 patients received a form of psychotherapy (including conversations with a psychologist/psychiatrist, psychotherapy, Eye Movement Desensitization and Reprocessing, systemic therapy or group therapy) within the month before baseline. Two of those received cognitive behavioral therapy. Four patients did not receive any psychotherapy or pharmacological treatment.

**Table 1:** Demographic and clinical information of the subject group at baseline and for both, the positive and negative outcome group at 3-months and 6-months follow-up. Normality of distributions was tested using a Shapiro-Wilk test.

	**Diagnosis**			**Outcome (at 6-months)**		
Variable \ outcome	*HC*	*MDD*	*p*	*Positive outcome*	*Negative outcome*	*p*
**n**	31	32	−	12	19	−
**Age (years)**	40.4 ± 15.5	43.8 ± 13.4	0.37^b^	40.3 ± 14.4	46.8 ± 12.3	0.19^a^
**Female/Male**	20 / 11	20 / 12	0.75^χ^	9 / 3	11 / 8	0.078^χ^
**Education level (1**–**5)**	1.94 ± 0.80	1.50 ± 0.88	0.06^b^	1.67 ± 0.98	1.31 ± 0.75	0.25^b^
**HDRS score ^c^**	0.67 ± 1.37	24.4 ± 4.62	0.00^***^^a^	10.8 ± 3.36	18.7 ± 4.66	0.00^**^^a^
**n lifetime MDD episodes**	−	2.03 ± 0.69	−	1.75 ± 0.62	2.26 ± 0.65	**0.043^b^**
**Duration current episode (months)**	−	11.5 ± 6.33	−	10.3 ± 6.69	12.1 ± 6.21	0.45^a^
**First MDD onset (years)**	−	33.8 ± 13.4	−	32.5 ± 14.8	34.9 ± 13.1	0.56^b^

^a^ p-value calculated from a two-sample *t*-test (normal distribution); ^b^ p-value calculated from a Mann-Whitney *U* test (non-normal distribution); ^c^ HDRS score at baseline is shown for the diagnosis group and HDRS score at 6-months follow-up is shown for the outcome group; ^χ^ p-value calculated from a χ^2^ test. Abbreviations: HDRS = Hamilton Depression Rating Scale; HC = healthy control; MDD = major depressive episode.

Participants were selected based on several selection criteria. The inclusion criterium for both groups was (I) age between 18 and 65 years old. For the MDD group, an additional inclusion criterium was (II) a diagnosis of unipolar MDD (according to the Diagnostic and Statistical Manual of Mental Disorders version 5 (American Psychiatric Association, 2013); DSM-5) as assessed through the Mini International Neuropsychiatric Interview by board-certified psychiatrists (van Vliet and de Beurs, 2007). Exclusion criteria for all participants were (I) any concurrent neurological or psychiatric disorder; (II) current substance or alcohol abuse; (III) a history of psychosis, autism spectrum disorder, attention deficit hyperactivity disorder or (mild) intellectual disability; (IV) any contra-indication for MRI (non-compatible brain MRI tattoos or implants, pregnancy, claustrophobia); (V) previous or current treatment with electroconvulsive therapy, deep-brain stimulation or transcranial magnetic stimulation. Additional exclusion criteria specifically for MDD were (VI) more than 3 previous MDD episodes (excluding the current one); (VII) a current MDD episode lasting longer than 2 years. An additional exclusion criterium for the HC group was (VIII) a current or previous episode of MDD according to the DSM-5.

All participants gave written informed consent to participate voluntarily in this study minimally a week after being informed about the study procedures. The study was approved (NL73949.015.20) by the Medical Ethical Review Commission Maxima Medical Centre, Veldhoven, the Netherlands (W20.054) on September 28th in 2020. The clinical study is registered at clinicaltrials.gov with identifier NCT05701267.

### 2.2 Clinical assessments

The depression severity was assessed with the Hamilton Depression Rating Scale (HDRS) 17-items (Hamilton, 1960) at the beginning of the study (first visit at the investigational site, further on referred to as baseline) and 6-months after baseline. This questionnaire assesses various symptoms associated with MDD and ranges between 0 and 52, where a higher value indicates a more severe depressive episode. The relative change in HDRS from follow-up to baseline was calculated to divide participants into groups of PO or NO as later explained in Section 2.6 “Classification”.

Participants received treatment as usual, i.e. by prescription of their external clinical expert (psychologists or psychiatrists), or no treatment. That is, in this study we did not alter their treatment plan, referred to as a naturalistic setting. Treatment of MDD subjects varied between psychotherapy, pharmacotherapy, a combination thereof or no treatment at all.

### 2.3 MRI acquisition

MRI scans were acquired with a Philips 3 T Achieva dStream (Philips Healthcare, Best, the Netherlands) at the Expertise Centre For Epilepsy and Sleep Disorders Kempenhaeghe (Heeze, the Netherlands). The following MRI modalities were obtained: T_1_-weighted, high-resolution T_2_-weighted (field-of-view of the amygdala and hippocampus), resting-state and task-based fMRI, and DTI. Scan parameters can be found in Supplementary Materials 1.1. External cardiac and respiratory signals were obtained using a photoplethysmographic unit placed on a finger and a respiratory belt placed on the abdomen, respectively.

An adapted version of the Hariri task was performed during the task-based fMRI scan to assess emotion processing (Hariri et al., 2002). Participants were instructed to match shapes or angry/fearful faces with a target using a button press. In total, there were 7 blocks of rest, 6 blocks of shapes, and 6 blocks of faces, each lasting 27 s (20 volumes). Stimuli were presented for 3 s with an inter-stimulus interval of 1 s. See Supplementary Figure S1.

### 2.4 MRI processing

This section highlights the most crucial processing steps performed to denoise the MRI data before extraction of the classification input features. More details can be found in Supplementary Materials Section 1.2.

T_1_-weighted brain (sub)region volumes and cortical thickness measures were obtained using Freesurfer (https://surfer.nmr.mgh.harvard.edu/fswiki/recon-all). Volumes of hippocampal subfields and amygdalar nuclei were extracted from the high-resolution T_2_-weighted scan and T_1_-weighted scan (Iglesias et al., 2016, Iglesias et al., 2015, Saygin et al., 2017).

fMRI preprocessing was performed using Statistical Parametric Mapping software (SPM12, https://www.fil.ion.ucl.ac.uk/spm/, RRID:SCR_007037) and additional functions from the FMRIB Software Library v6.0 (FSL, RRID:SCR_002823) package (Jenkinson et al., 2012). In short, initial steps included slice-timing correction and realignment. Then, ‘optimal’ multi-echo combination was applied (Posse et al., 1999) to combine the fMRI data acquired at different TEs. Subsequently, the T_1_-weighted scan was co-registered to the fMRI scan, and segmented into separate tissue classes including white and gray matter (WM;GM) and cerebral spinal fluid. Images were spatially normalized to Montreal Neurological Institute space. This was followed by regression of confounding time-series from WM and cerebral spinal fluid, bandpass filter between 0.01 and 0.2 Hz cutoffs (Pilmeyer et al., 2023) and spatial smoothing with a 5 mm full-width at half-maximum kernel.

Functional resting-state brain networks (RSNs) were extracted using group independent component analysis (ICA) with the Group ICA of fMRI Toolbox (GIFT v3.0c, https://icatb.sourceforge.net/, RRID:SCR_001953. A number of 35 ICs was found to be the optimal number of components to not split RSNs into subnetworks or obtain merged RSNs within a single component (Wang et al., 2011). 15 RSNs were identified (Supplementary Figure S2) from the 35 ICs based on a goodness-of-fit approach (Bernas et al., 2018) between ICs and the RSN atlas of Smith et al (Smith et al., 2009). Visual inspection was used to verify the automated identification process. These included the primary visual network (pVN), lateral visual network (latVN), occipital visual network (oVN), default mode network (DMN), cerebellum network (CN), sensorimotor network (SMN), auditory network (AN) and left and right frontoparietal network (l/rFPN). The DMN was further separated into the whole, anterior and posterior components (DMN, aDMN and pDMN), and the SMN also contained a lateral SMN (latSMN) component. Visual inspection identified 3 additional networks that are not part of the Smith et al. atlas: the salience network (SN), basal ganglia network (BN) and dorsal attention network (DAN) were identified based on previous RSN studies (Bharti, K., Graham, S.J., Benatar, M., Briemberg, H., Chenji, S., Dupré, N., Dionne, A., Frayne, R., Genge, A., Korngut, L., Luk, C., Zinman, L., Kalra, S., Consortium (CALSNIC), for the C.A.N., 2022, Mueller et al., 2014, Shi et al., 2020, Sorg et al., 2013).

DTI images were processed using MRtrix3 (Tournier et al., 2019) and FSL (Jenkinson et al., 2012). Images were denoised and corrected for distortions. Non-brain tissue was removed, and fiber orientation distribution maps were estimated through multi-shell, multi-tissue constrained spherical deconvolution (CSD) (Jeurissen et al., 2014). Following generation of a GM-WM boundary mask, anatomically constrained tractography (ACT) was applied to ensure streamlines were anatomically and biologically plausible (Smith et al., 2012). A total of 20 million streamlines was generated after which weights were assigned to each streamline (SIFT2) (Smith et al., 2015). These weights are based on minimizing the difference between the streamline density and the estimated fiber density from the diffusion model to reflect the underlying biological connectivity (Smith et al., 2022).

### 2.5 Feature extraction

A total of 11 different feature sets was extracted from the different MRI modalities. This section describes the extraction thereof. An overview of the feature sets and the number of feature elements can be found in Supplementary Table S1. More details and motivations can be found in Supplementary Materials Section 1.3.

#### 2.5.1 T_1_-weighted

Volumes of 43 subcortical structures and other brain regions (Fischl et al., 2002), and the cortical thickness of 68 cortical structures were obtained (Desikan et al., 2006). Thus, a total of 113 elements were obtained for the T_1_-based feature set.

#### 2.5.2 T_2_-weighted

The volumes of 44 hippocampal subfields and 20 amygdalar nuclei were extracted (Iglesias et al., 2016, Iglesias et al., 2015, Saygin et al., 2017), resulting in a total of 66 elements.

#### 2.5.3 DTI

Five measures of structural connectivity matrices were obtained from white matter tracts between 84 subcortical and cortical regions (Desikan et al., 2006, Fischl et al., 2002). Mean diffusivity (MD), FA, axial diffusivity (AD) and radial diffusivity (RD) and sum of streamline weights (sSW) were obtained for each unique connection, resulting in 3486 elements for each of the 5 DTI features. The sSW feature is a more biologically accurate measure of white matter density (Smith et al., 2015).

#### 2.5.4 Task-based fMRI

From general linear models including regressors for each condition and several confounders, activation contrast maps (t-values) were obtained between the Faces > Shapes and Faces > Rest conditions. The average contrast was calculated for 11 ROIs and the 2 pairs of conditions, resulting in 22 elements.

#### 2.5.5 Resting-state fMRI

Static FC was calculated between each unique pair of RSNs, resulting in 105 elements. Furthermore, wavelet coherence analysis (WCA) was performed, which assesses the temporally changing interactions between RSNs. Two features were derived from WCA: lead coherence (leadCoh) and the number of coherence clusters (nCC) as described by Cîrstian et al. using the same parameters (Cîrstian et al., 2023). LeadCoh reflects a causal effect between pairs of RSNs, i.e. RSN 1 leads in phase compared to RSN 2. nCC describes the amount of different interactions (clusters) that occur between RSN pairs during the resting-state fMRI scan, including the time-series being in-phase, out-of-phase, leading or lagging. A total of 105 and 210 elements were extracted for nCC and leadCoh, respectively (leadCoh matrix is not symmetrical).

### 2.6 Classification

#### 2.6.1 Uni-modal

Classification was performed for the diagnostic and outcome case and for each MRI feature set. For the diagnostic classifications, models aimed to distinguish MDD participants from HCs. For the outcome, participants were divided into two classes based on change in symptom severity. This was done by calculation of the relative change in HDRS score at the 6-months follow-up compared to baseline. If a participant showed a reduction of ≥ 50 % in depression severity compared to baseline, it was labeled as PO. Alternatively, the participant was labeled as NO. This cutoff of relative change in HDRS has been accepted and validated as the golden standard for evaluation of clinically significant improvement (Bobo et al., 2016, Lacroix et al., 2021). Out of the 32 MDD patients, one decided to stop the study before the follow-up. The 31 other MDD patients completed the 6-months follow-up and were divided into 12 PO (age 40.3 ± 14.4 years, 3/9 male/females) and 19 NO (age 46.8 ± 12.3 years, 8/11 male/females) participants, see Table 1.

An SVM classifier with a linear kernel was implemented using leave-one-out nested cross-validation. The inner loop of the nested cross-validation was used for feature selection on the n-1 subjects. The only parameter that was optimized was the number of features. For each fold in the outer loop, the optimal number of features (determined by the highest AUC over all inner loop iterations) was used.

Because several feature sets had a high number of feature elements, making the task compute-intensive and the comparison between the feature sets biased, double feature ranking was applied (Drenthen et al., 2021, Yang et al., 2021). Ranking was applied on the training set whereas the left-out test sample remained untouched until performance testing. First, the k_initial_ most significant features elements were selected based on group differences. This was performed with a two-sample *t*-test or Wilcoxon rank sum test, depending on normality of the group distributions as assessed with the Shapiro-Wilk test (Mann and Whitney, 1947, Shapiro and Wilk, 1965). After initial selection, the k_initial_ left-over feature elements were added one-by-one, starting with the highest ranked feature elements, adding the next one and iterating until a k_final_ maximum number of features. Two feature ranking methods were applied:

1) Minimum redundancy maximum relevance (MRMR, (Ding and Peng, 2005)): higher feature ranking corresponds to features where mutual information is maximized (maximum relevance) between a feature and the response variable while reducing the mutual information (minimum redundancy) between this feature and other features. MRMR with a linear SVM classifier based on DTI and fMRI features have been successfully (78 % and 86 % accuracy, respectively) applied to objectively classify MDD before (Chu et al., 2018, Cîrstian et al., 2023).
2) Concave minimization and support vector machines (CV-SVM, (Mangasarian and Bradley, 1998)): Two bounding planes are calculated between the feature sets of the two classes. The separating plane is then calculated by minimizing the sum of distances of incorrectly classified points between the bounding plane. Furthermore, the dimensionality of features is reduced as much as possible and additional SVM maximizes the distance between the two bounding planes. CV-SVM feature selection in combination with a linear SVM and resting-state fMRI features has been shown to discriminate HCs from patients with schizophrenia with > 97 % accuracy (Algumaei et al., 2022)

From the k_final_ feature elements, the optimal number of features elements, k_optimal_, was determined based on the highest validation AUC. After k_optimal_ was determined from the inner loop, the same initial selection and final ranking procedures were applied to the training set of the outer loop. The k_optimal_ highest ranked features from the training set of the outer loop were then applied to the unseen data from the test subject to predict the test label. The whole procedure was repeated for each outer loop fold. Performance was reported for the validation and test sets: AUC, accuracy, sensitivity, specificity, f1-score and precision.

Different combinations of cutoff values for the maximum number of features were tested for the initial group difference based selection and second round of feature selection. Ranges were the following: k_initial_ ∈ {5, 10, 20, 50, 100, All} and k_final_ ∈ {3, 5, 10, 20}. Only range combinations that were possible were run (e.g. k_initial_ = 10 and k_final_ = 20 is not possible). We report here the findings of the combination k_initial_ = 10 and k_final_ = 5 since these provided the highest overall AUC.

To test significance of each model, i.e. determine whether the model performed significantly above chance level (AUC = 0.50), permutation tests were conducted (Bhaumik et al., 2017). Through permutation of the class labels while maintaining the data, a null distribution was created. The AUC of each model was then compared with the obtained null distribution. The whole classification set-up was maintained (nested cross-validation with double feature selection) and the labels were permutated 1000 times (Bhaumik et al., 2017). The p-value was obtained by counting the number of times the permutation AUC exceeded the AUC true label test and dividing it by 1000. A p-value ≤ 0.05 was considered as significant.

Furthermore, to assess the uncertainty of the AUC values, 95 % confidence intervals were calculated. For each reported AUC value, bootstrapping was performed. The subjects’ data were randomly resampled 1000 times with replacement, i.e. subjects could be selected multiple times in a single bootstrap sample. Each bootstrap sample contains the same number of subjects as in the original sample (i.e. n = 63 for diagnosis and n = 31 for prognosis). From each of the 1000 bootstrap samples, the AUC was calculated over the new set of predicted labels. Finally, the 95 % confidence interval was calculated over the 1000 AUC values, obtained from the 1000 bootstrap samples. Confidence intervals (CI) are reported as [CI_min_, CI_max_].

### 2.7 Multi-modal

To assess the effect of combination of modalities, three different methods were tested:

1) Concatenation: All feature sets were concatenated to one feature vector and the same procedure as for the uni-modal classification was applied.
2) Forced fusion: From each feature set, the top 5 feature elements were selected by initial selection based on group differences in the training sets of each inner fold (similar to the method to determine k_initial_, as described in the uni-modal section). This ensures that feature elements from all feature sets were selected in the initial multi-modal feature set. Subsequently, MRMR or CV-SVM feature ranking was applied where k_optimal_ was obtained in the same way as in the uni-modal approach with k_final_ = 5.
3) Ensemble classifier: The predicted labels from the SVM classifiers in the uni-modal approach were subjected to majority voting in order to obtain an ensemble prediction. Several ensemble classifiers were created, including all 11 feature sets and all compossible combinations with n_set_ ∈ {3, 5, 7, 9}.

### 2.8 Feature analysis

To assess which feature elements had the most impact on prediction of MDD and its outcome, the percentage that a feature element was present in the outer folds was calculated. We perform these feature contribution analyses on each optimal uni-modal model.

For analysis of the impact of features on the performance using the most optimal (highest AUC) multi-modal model, an ablation experiment was conducted. In each iteration, a feature set was removed from the multi-modal model to assess the reduction in AUC. Features that result in a higher reduction of AUC are associated with a higher impact on the multi-modal model.

Furthermore, an explorative group difference analysis was performed on all feature elements. For each feature set, group differences between MDD and HC groups or between PO and NO groups were calculated based on either the two-sample *t*-test or Wilcoxon rank sum test, depending on normality of the group distributions as assessed with the Shapiro-Wilk test. For each feature set, p-values were corrected for multiple comparisons using the false discovery rate (FDR) with α = 0.05 (Benjamini and Hochberg, 1995).

## 3 Results

### 3.1 Classification

#### 3.1.1 Uni-modal

**Fig. 2:**
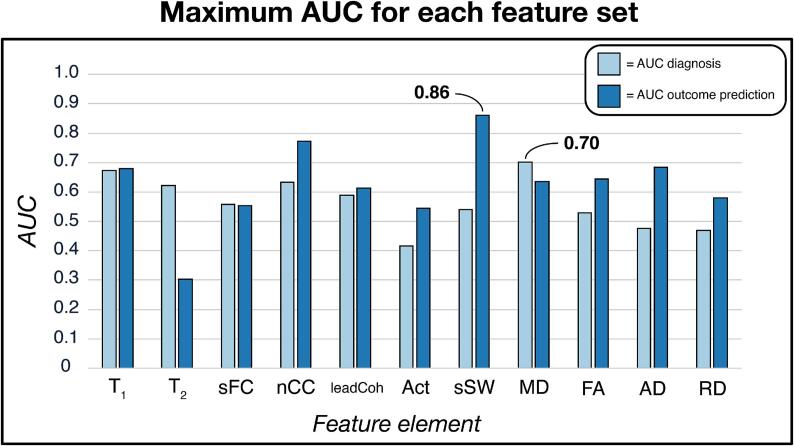
**Uni-modal classification performance** The area under the curve (AUC) metric is calculated for each uni-modal feature set. This is shown for classification between subjects with major depressive disorder versus healthy controls (diagnosis; light blue) and for positive versus negative outcome at 6-months follow-up (outcome prediction; dark blue). For diagnosis, the highest AUC was 0.70 based on the mean diffusion (MD) feature set. For outcome prediction, the most optimal model resulted in an AUC of 0.86 based on the sum of streamline weights (sSW) feature set. Abbreviations: sFC = static functional connectivity; nCC = number of coherence clusters; Act = activity; FA = fractional anisotropy; AD = axial diffusivity; RD = radial diffusivity.

AUC performance for the most optimal SVM models of each feature set and the diagnostic and classifications can be found in Fig. 2 and all other metrics in Supplementary Table S2. The corresponding validation performance of these optimal models can be found in Supplementary Table S3. For diagnosis, the AUC was the highest for the MD feature set (0.701 [0.699, 0.706], p < 0.01) with a sensitivity of 71.9 % and specificity of 67.7 %. For outcome, the sSW yielded the highest AUC (0.860 [0.858, 0.866], p < 0.001). Performance metrics were relatively imbalanced with a sensitivity of 91.7 % and specificity of 68.4 %. The nCC yielded the highest AUC overall (p < 0.05 and p < 0.01 for diagnosis and outcome prediction, respectively).

#### 3.1.2 Multi-modal

**Fig. 3:**
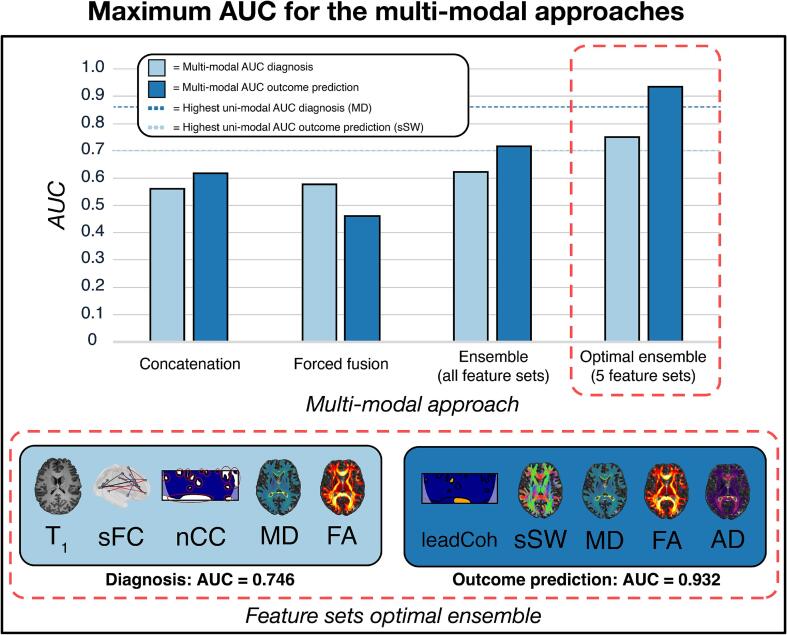
**Multi-modal classification performance** The area under the curve (AUC) metric is calculated for each multi-modal feature set. This is shown for classification between subjects with major depressive disorder versus healthy controls (diagnosis; light blue) and for positive versus negative outcome (outcome prediction; dark blue). For diagnosis, a multi-modal ensemble of 5 feature sets (T_1_-weighted, sFC, nCC, MD and FA) improved the AUC to 0.746 (highest uni-modal AUC = 0.701). For outcome predictiion, a multi-modal ensemble of 5 feature sets (leadCoh, sSW, MD, FA and AD) increased the AUC to 0.932 (highest uni-modal AUC = 0.860). Abbreviations: sFC = static functional connectivity; nCC = number of coherence clusters; MD = mean diffusivity; FA = fractional anisotropy; leadCoh = lead coherence; sSW = sum of streamline weights; AD = axial diffusivity.

While multi-modal concatenation of the feature vectors and forced fusion of the top 5 feature elements from each feature set did not result in improvement, ensemble models based on 5 feature sets achieved improved AUC for both diagnosis and outcome prediction compared to uni-modal models, see Fig. 3 and Supplementary Table S4. For diagnosis, an AUC of 0.746 [0.741, 0.747] (sensitivity 78.1 % and specificity 71 %) was achieved with an ensemble model based on T_1_, sFC, nCC, MD and FA features. For outcome prediction, an ensemble model based on leadCoh, sSW, MD, FA and AD yielded an AUC of 0.932 [0.927, 0.934] (sensitivity 91.7 % and specificity 94.7 %). Moreover, several other multi-echo ensembles resulted in improved performance compared to a uni-modal approach, see Supplementary Table S5. A total of 11 and 17 ensemble models (of 3, 5 or 7 features) improved the top uni-modal for diagnosis and outcome, respectively. Of these 28, only 1 ensemble model consisted of a feature set combinations with measures from a single modality. Of all ensemble models that improved AUC compared to a uni-modal approach, 7.1 % consisted of feature sets with 2 modalities, 46.4 % with 3 modalities, 39.3 % with 4 modalities and lastly there was one ensemble model that contained features from all 5 modalities acquired in this study.

### 3.2 Feature analysis

#### 3.2.1 Uni-modal feature contribution

**Fig. 4:**
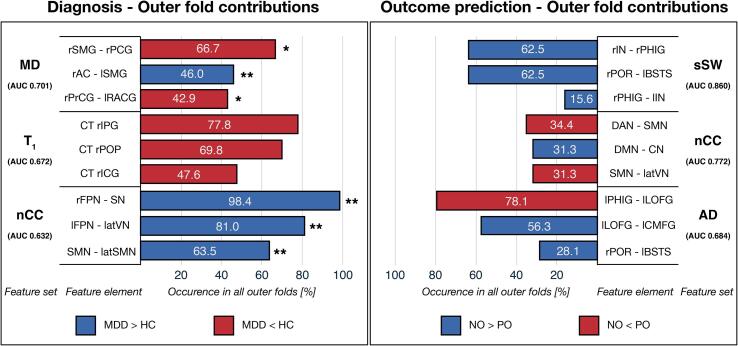
**Highest feature contributions** Feature contribution is calculated by occurence in the test models of the outer folds (percentage) for the top 3 feature sets and elements. Colors indicate increases and decreases between groups. The symbols *, **, and *** indicate uncorrected p-values ≤ 0.05, 0.01, and 0.001, respectively. Abbreviations: MDD = major depressive disorder; HC = healthy controls; NO/PO = negative/positive outcome; AUC = area under the curve; MD = mean diffusivity; nCC = number of coherence clusters; sSW = sum of streamline weights; AD = axial diffusivity; r = right; l = left; lat = lateral; SMG = supramarginal gyrus; PCG = posterior cingulate gyrus; AC = accumbens area; PrCG = precentral gyrus; RACG = rostral anterior cingulate gyrus; IPG = inferior parietal gyrus; POP = pars opercularis; ICG = isthmus cingulate gyrus; FPN = frontoparietal network; SN = salience network; VN = visual network; SMN = sensorimotor network; IN = insula; PHIG = parahippocampal gyrus; POR = pars orbitalis; BSTS = bank of superior temporal sulcus; DAN = dorsal attention network; DMN = default mode network; CN = cerebellum network; LOFG = lateral orbitofrontal gyrus; CMFG = causal middle frontal gyrus.

The 3 highest feature element contributions for the top 3 uni-modal feature models in diagnosis and outcome prediction are shown in Fig. 4. For an overview of all features, see Supplementary Table S6 and S7.

For diagnosis, the highest contribution in the outer folds for each of the top 3 feature sets: increased mean diffusivity between the right supramarginal gyrus and right posterior cingulate gyrus (66.7 %), decreased cortical thickness of the inferior parietal gyrus (77.8 %) and increased nCC between the right frontoparietal network and the salience network (98.4 %). Another interesting trend can be observed in the T_1_ and nCC feature sets. The top 3 feature elements of T_1_ set all display decreased right hemisphere cortical thickness in MDD and the nCC network pairs are all increased.

For outcome prediction, the feature elements with the highest outer fold contribution for the top 3 feature sets comprise: increased sSW between the right insula – right parahippocampal gyrus and the right pars orbitalis − left banks of superior temporal sulcus and between (both 62.5 %), increased nCC between the default mode network and the sensorimotor network (34.4 %) and increased AD between the left parahippocampal gyrus and the left lateral orbitofrontal gyrus (78.1 %) in the NO group compared to the PO group. Interesting observations include an increase in sSW in the top 3 feature elements for the NO group and high contributions from the parahippocampal gyri (top feature element in sSW and AD).

As the feature contribution is more related to feature occurrence, we also calculated the feature impact on the models by assessment of the β-coefficients that form the hyperplane of the linear SVMs. The higher a β-coefficient, the more effect on the hyperplane and thus classification outcome. Again we calculated the top 3 elements for each feature, see Supplementary Table S8 and S9. In general, the top elements for feature impact were relatively similar to feature contribution. However, it can be observed that the most impactful feature for outcome prediction based on sSW is the connection between the right insula and left superior temporal gyrus. This finding, together with the feature contribution, indicates the importance of the connections with the insula. For diagnosis, there were no significant changes in the top feature elements.

### 3.3 Multi-modal feature contribution

**Fig. 5:**
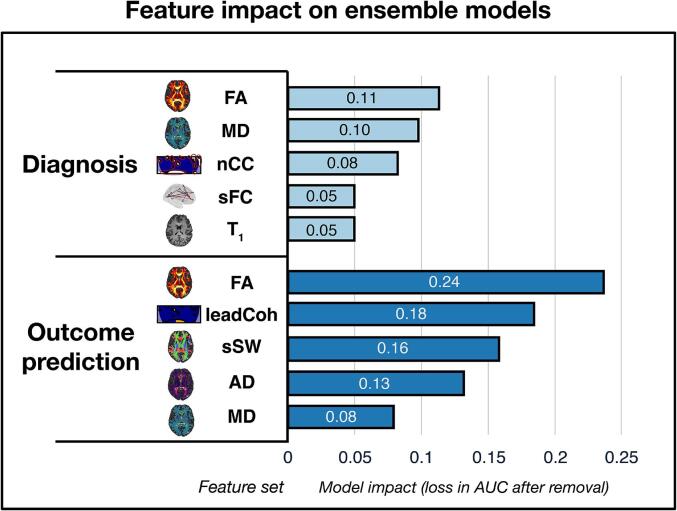
**Ablation experiment results** The ablation experiment demonstrates the impact of each feature set on the performance of the optimal multi-modal ensemble models. For each feature set, impact is measured as loss in area under the curve (AUC) after removal from the ensemble model. Abbreviations: FA = fractional anisotropy; MD = mean diffusivity; nCC = number of coherence clusters; sFC = static functional connectivity; leadCoh = lead coherence; sSW = sum of streamline weights; AD = axial diffusivity.

The results of the ablation experiment to assess the contribution of each uni-modal feature set in the top multi-modal ensemble model for diagnosis and outcome prediction can be found in Fig. 5.

In both diagnosis and prognosis, FA impacted the performance of the most optimal multi-modal ensemble model the most. Removal of the FA feature set in the ensemble resulted in an AUC loss of 0.113 (from 0.746) and 0.237 (from 0.932) for diagnosis and outcome prediction, respectively. For both ensemble models, two DTI measures and one fMRI wavelet measure were among the top 3 feature set contributions.

**Fig. 6:**
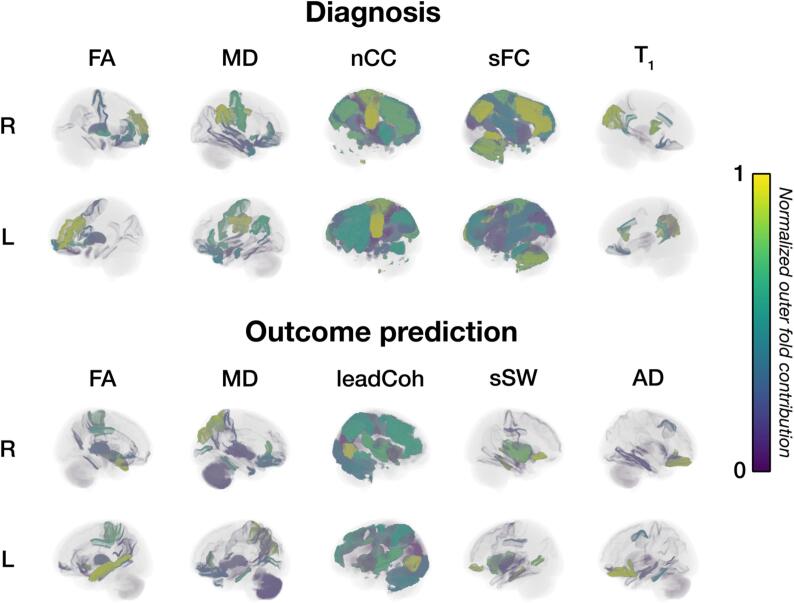
**Brain areas of key predictors** The brain visualizations represent areas that resulted in the highest multi-modal ensemble performance for diagnosis and outcome prediction. The five feature sets comprising each optimal ensemble model are displayed. Colors indicate normalized outer fold contributions. Normalization was performed by taking the minimum and maximum outer fold contributions over all models to allow comparison between the models. The gray brain background indicates that these regions or networks were not part of the feature set of that model. Abbreviations: R = right; L = left; FA = fractional anisotropy; MD = mean diffusivity; nCC = number of coherence clusters; sFC = static functional connectivity; leadCoh = lead coherence; sSW = sum of streamline weights; AD = axial diffusivity.

To further visualize the affected areas in the brain, the outer fold contributions of each of the five feature sets from both ensemble models were normalized and plotted in the brain, see Fig. 6. Generally, it can be observed that the highest contributing regions were found in frontal and limbic areas whereas networks were mostly located in frontoparietal areas. While the most optimal ensemble classifier of diagnosis and outcome prediction both comprise the FA and MD, the brain areas that distinguish between the groups were different. For example, the model with the highest AUC for FA in diagnosis classification was mostly based on frontolimbic areas (rostral middle frontal gyrus, rostral anterior cingulate gyrus, pallidum and frontal pole) whereas the areas supporting the top FA models in outcome prediction were more widespread (superior temporal gyrus, accumbens area and paracentral gyrus). For diagnosis, highly contributing areas in the MD models were more widespread (posterior cingulate gyrus, supramarginal gyrus and precentral gyrus) whereas those for outcome prediction were located mostly in the parietal and limbic lobe (superior parietal gyrus, rostral anterior cingulate gyrus and parahippocampal gyrus).

### 3.4 Group difference analysis

While 745 feature elements were significant when uncorrected in MDD versus HC participants, 0 remained significant after FDR correction. Of the 1222 significant feature elements between the NO and PO group, only 2 remained significant. The activity contrast (t-value) in the anterior cingulate cortex (ACC) between face and rest conditions was higher in the NO group (NO: −2.02 • 10^-1^ ± 7.36 • 10^-1^; PO: −1.30 • 10^0^ ± 1.20 • 10^0^) and in the subgenual ACC (NO: −1.38 • 10^-1^ ± 9.88 • 10^-1^; PO: −1.28 • 10^0^ ± 9.93 • 10^-1^). The top 10 most significant (lowest p-value) feature elements for diagnosis and outcome groups can be found in Supplementary Table S10.

## 4 Discussion

In this paper we sought to diagnose MDD and predict MDD outcome objectively using different MRI modalities. Additionally, we aimed to evaluate the potential benefits of combining multiple modalities to improve classification performance. Through a uni-modal approach, MDD subjects were distinguished from HCs with an AUC of 0.701 based on MD, whereas 6-months outcome groups were separated with an AUC of 0.860 based on sSW. A multi-modal ensemble classifier of 5 features, derived from T_1_, resting-state fMRI and DTI scans, both improved objective diagnosis (AUC 0.746) and outcome prediction (AUC 0.932). Generally, feature analysis revealed that predominantly frontolimbic regions and frontoparietal networks were most contributing to the diagnosis and outcome prediction. However, the brain location of the most valuable features were found to be dependent on the feature and the model (diagnosis versus outcome prediction), highlighting the fact that MDD diagnostic biomarkers may be different than biomarkers predicting outcome. These findings confirm our hypotheses that multi-modal imaging improves classification performance in MDD and MDD outcome.

### 4.1 Uni- versus multi-modal

For the uni-modal approach, several trends were noticeable. First, the highest AUC was obtained by two DTI measures: MD for diagnosis (AUC 0.701) and sSW for outcome prediction (AUC 0.860). These results show the promise of the relatively novel DTI acquisition (multiband, 96 directions and 3 shells) and processing (CSD, ACT and SIFT2) which allow DTI measures to be assessed along inter-regional WM tracts while correcting for bias in fiber-tracking algorithms, providing a more biologically accurate measure of structural connectivity. Second, overall diagnostic performance was lower but more balanced (sensitivity versus specificity) overall compared to outcome prediction performance. Third, the number of optimal feature elements was relatively stable, with a mean around 2.5 to 3.0. The stability of k_optimal_ may be the result of the implementation of a nested cross-validation, allowing to tune the optimal number of feature elements independently of the left-out test sample. By separating model selection and evaluation in an inner and outer loop, nested LOOCV assesses the generalization performance. This is crucial for model performance on unseen data, e.g. from follow-up studies using different datasets and patients.

Multi-modal ensemble models improved both diagnosis (highest AUC 0.746) and outcome prediction performance (highest AUC 0.932). In total, 11 diagnostic and 17 outcome ensemble models (with 3, 5 or 7 feature sets) improved the AUC of the highest uni-modal approach. Of note is the high balance between sensitivity and specificity for both diagnostic and outcome prediction models. For diagnosis, sensitivity and specificity ranged between 62.5–78.9 and between 67.7–80.6, respectively. For outcome, this ranged between 75.0–100 and 84.2–100. This is in agreement with other MRI studies that implemented ensembles of linear SVM classifiers (Pei et al., 2020, Varol et al., 2012).

### 4.2 Diagnosis versus outcome prediction

Different features were found to be most important for diagnostic versus outcome prediction. For diagnosis, nCC, sFC and T_1_ were part of the most optimal ensemble models whereas leadCoh, sSW and AD were most contributing to the outcome prediction models. Furthermore, where a general consensus was found that the highest discriminating areas between MDD and HCs and the two MDD outcome groups were located in frontal, parietal and limbic areas of the brain, the specific regions or networks were different between the two pairs of classes.

Even in the case of FA and MD, which were found in both ensemble models, the most important regions were located in different parts of the brain for diagnosis versus outcome prediction: the model with the highest AUC for FA in MDD classification was mostly based on frontolimbic areas (rostral middle frontal gyrus, rostral anterior cingulate gyrus, pallidum and frontal pole) whereas the areas supporting the top FA models in outcome prediction were more widespread (superior temporal gyrus, accumbens area and paracentral gyrus). Regarding MD, high diagnostic model contributions were found in more widespread areas (posterior cingulate gyrus, supramarginal gyrus and precentral gyrus) whereas those in outcome prediction were located mostly in the limbic and parietal lobe (superior parietal gyrus, rostral anterior cingulate gyrus and parahippocampal gyrus).

Thus, while FA and MD may both be valuable for MDD classification and prediction of MDD outcome, the affected brain location and direction of change differ. This may be crucial for future prognostic biomarker studies which aim to define ROIs a-priori or hypotheses based on findings of previous studies reporting changes in MDD versus HCs (or vice versa). These predefined ROIs or hypotheses may not translate to similar findings between outcome groups.

### 4.3 Clinical interpretation

In general, the most important structural features were found to be located in frontolimbic regions. MDD-related frontolimbic alterations in brain structure and function have been reported before (Arnold et al., 2012, Lai, 2021, LeWinn et al., 2014). This circuitry is associated with emotion regulation (Kebets et al., 2021). Moreover, we found mostly features based on functional networks to be located in frontoparietal areas. Alterations in these brain regions have been associated with cognitive dysfunction, and rumination (Lai, 2019).

More specifically, as reflected by the ablation study, FA was the most important feature for diagnostic and prognostic multi-modal models. For diagnosis, the connections in frontolimbic areas (rostral middle frontal gyrus, rostral anterior cingulate gyrus, pallidum and frontal pole) yielded the highest feature importance. For outcome prediction, these were more widespread (superior temporal gyrus, accumbens area and paracentral gyrus). A previous *meta*-analysis on DTI in MDD versus HCs found decreases in FA of the corona radiata (van Velzen et al., 2019), large bundles of fibers connecting with the ACC and frontal regions (Jiang et al., 2019). These findings match with our results where FA in tracts of the ACC and several frontal regions were decreased. The frontal part of the corona radiata is part of the limbic-thalamo-cortical circuit and reduced WM integrity thereof may affect cognitive and emotional regulation (Sanjuan et al., 2013). Another finding that agrees on existing literature regards the increase in FA in in the NO group between the tracts connecting the accumbens area – posterior cingulate cortex (PCC). The accumbens area, part of the ventral striatum, is a target area for deep brain stimulation in MDD (Figee et al., 2022). This circuitry overlaps with the reward-circuitry and therefore has been linked to anhedonia, a common symptom in MDD. Moreover, hyperconnectivity between the ventral striatum and the posterior DMN (including the PCC) in MDD subjects has been shown to normalize following electroconvulsive therapy (Leaver et al., 2016). Perhaps the NO group in our study, with higher FA in this tract and not responding to psychotherapy and/or pharmacotherapy, may therefore better respond to more invasive treatments.

For single-modal models, MD and sSW yielded the highest performance for diagnosis and outcome prediction, respectively. For diagnosis, these were located in, amongst others, the posterior cingulate gyrus, supramarginal gyrus and precentral gyrus. While we did not find previous reports of decreased MD surrounding the PCC, it is known to be the key hub of the DMN (Fransson and Marrelec, 2008). Altered functional connectivity of the DMN has frequently been reported in studies, but the direction of change has been disputed (Pilmeyer et al., 2022). This finding of decreased MD of tracts connecting with the PCC in MDD patients may reflect increased structural connectivity. Enhanced activation of and connectivity with of the PCC hub has been associated with rumination in MDD (Hamilton et al., 2015, Zhou et al., 2020). For outcome prediction, sSW model contributions and β-coefficient analyses revealed the importance of white matter tracts connecting the insulae and parahippocampal gyrus. Together with the medial prefrontal cortex, these two regions form the paralimbic system, which is linked to emotional regulation, self-projection and memory (Jang et al., 2018). Moreover, decreases in functional connectivity between the insula and parahippocampal gyri have been associated before with negative emotion (Jang et al., 2018). Our finding of decreased white matter density (reflected by sSW) in connections to and from these regions may reflect similar abnormalities in MDD.

### 4.4 Study limitations and strengths

The current study has several limitations. First, outcome was defined binary and defined on change in severity estimated at two time points. Multi-class or continuous depression severity change prediction with regression models may ultimately be more useful in the clinic to select or change treatment for MDD patients. The course of MDD may vary over time: while some patients suffer from a one-time MDD episode, others may show more fluctuating or chronic trajectories. Thus, from a clinical viewpoint, future studies may add more practical value to models by taking this information into account.

Second, the relative change in HDRS with a 50 % cutoff has been validated as a measure of significant symptom improvement (Bobo et al., 2016, Furukawa et al., 2007, Leucht et al., 2013). However, it is rarely used as a sole instrument in clinical practice (Gautam et al., 2017, Ruhé et al., 2005). Other factors and measures, such as suicidality, malnutrition, comorbidities, safety, and costs often guide the treatment selection and adjustments (Gautam et al., 2017).

Third, the dataset was relatively small. Models from the current study may not generalize to other datasets and thus should still be tested on a separate dataset. Moreover, we explored a rich multi-modal MRI dataset derived in an observational longitudinal study setting, whereas multi-site studies often are limited to uni-modal acquisition in a cross-sectional design. Overfitting is also a greater risk with smaller datasets and a high number of features. However, in this study, we limited this by double and strict feature selection and nested LOOCV. Permutation tests indicated that the obtained classification performance was not obtained by chance since the true performance was significantly higher than the null distribution of the performance of the permutated models.

The study’s naturalistic setting may be considered as a limitation and strength. While this design does not determine the most effective treatment for a patient, it offers insight into how a patient’s symptoms may change given their current brain physiology. The strength is that a naturalistic design may be more generalizable or potentially more effective to real-world cases where patients have varied depression and treatment history. Moreover, there is only scarce evidence from naturalistic studies. We are only aware of one other treatment-as-usual MRI study in MDD (Schmaal et al., 2015). In this study, patients with a chronic trajectory could be discriminated from remitted patients with 73 % accuracy based on task-based fMRI features. After all, results from both types of studies may be required to identify robust predictive biomarkers in order to be used in the clinic.

This study also has several strengths. First, we performed classification to distinguish MDD patients from HCs and NO from PO groups using the same dataset. Because of this we were able to demonstrate that biomarkers may differ depending on the classification goal. Factors such as differences in selection criteria, acquisition parameters, image processing and analysis methods cannot explain the observed different features between diagnostic and outcome prediction models. Another advantage is the application of several novel MRI acquisition, processing and analysis methods. For example, multi-echo imaging in the fMRI scans has been shown to reduce signal loss and enhance the signal-to-noise ratio and quality of resting-state networks (Kundu et al., 2017, Pilmeyer et al., 2023). Multiband imaging reduces the scanning time which could be traded for improved spatial or temporal resolution in fMRI and DTI scans or to obtain more diffusion directions and shells. High-resolution (0.39 × 0.39 × 2.0 mm) T_2_-weighted scans were furthermore acquired which, in combination with T_1_-weighted scans allowed hippocampal subfield and amygdalae nuclei segmentation based on the novel Freesurfer tool. In addition, DTI measures were obtained along WM tracts instead of per voxel using the high angular multi-shell acquisition in combination with DCS and ACT. This alleviates the problem of crossing fibers and ensures DTI tracts are only accepted in more biologically plausible locations of the brain, reducing the amount of false positives. Besides that, we maintained relatively strict selection criteria, ruling out biases in results that may be explained by confounders such as comorbidities and invasive treatments that are known to have a significant impact on the brain. The downside of this choice is that it reduces generalizability. Since MDD is highly heterogeneous, and the treatments options are various, we set several boundaries to focus on a representative group of MDD patients (receiving no electrical stimulation treatment) without comorbidities to focus on the pathology of MDD itself.

## 5 Conclusions

In this work we aimed to identify MRI-based diagnostic and outcome biomarkers of MDD. We showed that DTI features were the most promising for diagnostic and prognostic models through a uni-modal approach. Multi-modal ensemble models with features derived from T_1_-weighted, resting-state fMRI and DTI scans improved the classification performance. These comprised relatively novel features, including functional network neurodynamics and more biologically accurate measures of structural connectivity. Crucially, we demonstrated that the brain areas of the most valuable features were different between diagnosis and outcome prediction. In general, the most valuable predictors were located in frontal, limbic and parietal regions. The current findings demonstrate the promise of multi-modal MRI for biomarker identification in MDD diagnosis and outcome prediction. Future work may focus on replication of the current classification pipeline with a larger and more heterogeneous dataset or on further exploration of more sophisticated approaches to define outcome.

## Funding sources

This work was supported by funding from the Top Consortia for Knowledge and Innovation Public-Private Partnerships (TKI-PPP, subsidy identification number TK11812P07) and additional funding from Philips.

## CRediT authorship contribution statement

**Jesper Pilmeyer:** Writing – review & editing, Writing – original draft, Visualization, Validation, Software, Methodology, Investigation, Formal analysis, Data curation, Conceptualization. **Rolf Lamerichs:** Writing – review & editing, Supervision, Software, Resources, Methodology, Data curation, Conceptualization. **Sjir Schielen:** Writing – review & editing, Methodology, Conceptualization. **Faroeq Ramsaransing:** Writing – review & editing, Methodology, Investigation. **Vivianne van Kranen-Mastenbroek:** . **Jacobus F.A. Jansen:** Writing – review & editing, Validation, Supervision, Methodology, Conceptualization. **Marcel Breeuwer:** Writing – review & editing, Validation, Supervision, Methodology, Conceptualization. **Svitlana Zinger:** Writing – review & editing, Supervision, Project administration, Methodology, Conceptualization.

## Declaration of Competing Interest

The authors declare that they have no known competing financial interests or personal relationships that could have appeared to influence the work reported in this paper.

## Data Availability

The authors do not have permission to share data.

## References

[b0005] Abe O., Yamasue H., Kasai K., Yamada H., Aoki S., Inoue H., Takei K., Suga M., Matsuo K., Kato T., Masutani Y., Ohtomo K. (2010). Voxel-based analyses of gray/white matter volume and diffusion tensor data in major depression. Psychiatry Res.

[b0010] Abi-Dargham A., Horga G. (2016). The search for imaging biomarkers in psychiatric disorders. Nat Med.

[b0015] Algumaei A.H., Algunaid R.F., Rushdi M.A., Yassine I.A. (2022). Feature and decision-level fusion for schizophrenia detection based on resting-state fMRI data. PLoS One.

[b0020] Arnold J.F., Zwiers M.P., Fitzgerald D.A., van Eijndhoven P., Becker E.S., Rinck M., Fernandez G., Speckens A.E.M., Tendolkar I. (2012). Fronto-limbic microstructure and structural connectivity in remission from major depression. Psychiatry Res.

[b0025] American Psychiatric Association, 2013. Diagnostic and Statistical Manual of Mental Disorders (DSM-5®). American Psychiatric Pub.

[b0030] Atkinson L., Sankar A., Adams T.M., Fu C.H.Y. (2014). Recent Advances in Neuroimaging of Mood Disorders: Structural and Functional Neural Correlates of Depression, Changes with Therapy, and Potential for Clinical Biomarkers. Curr Treat Options Psych.

[b0035] Benjamini Y., Hochberg Y. (1995). Controlling the False Discovery Rate: A Practical and Powerful Approach to Multiple Testing. Journal of the Royal Statistical Society. Series B (methodological).

[b0040] Bernas A., Aldenkamp A.P., Zinger S. (2018). Wavelet coherence-based classifier: A resting-state functional MRI study on neurodynamics in adolescents with high-functioning autism. Comput Methods Programs Biomed.

[b0045] Bharti, K., Graham, S.J., Benatar, M., Briemberg, H., Chenji, S., Dupré, N., Dionne, A., Frayne, R., Genge, A., Korngut, L., Luk, C., Zinman, L., Kalra, S., Consortium (CALSNIC), for the C.A.N. (2022). Functional alterations in large-scale resting-state networks of amyotrophic lateral sclerosis: A multi-site study across Canada and the United States. PLoS One.

[b0050] Bhaumik R., Jenkins L.M., Gowins J.R., Jacobs R.H., Barba A., Bhaumik D.K., Langenecker S.A. (2017). Multivariate pattern analysis strategies in detection of remitted major depressive disorder using resting state functional connectivity. Neuroimage Clin.

[b0055] Bobo W.V., Angleró G.C., Jenkins G., Hall-Flavin D.K., Weinshilboum R., Biernacka J.M. (2016). Validation of the 17-item Hamilton Depression Rating Scale definition of response for adults with major depressive disorder using equipercentile linking to Clinical Global Impression scale ratings: analysis of Pharmacogenomic Research Network Antidepressant Medication Pharmacogenomic Study (PGRN-AMPS) data. Hum Psychopharmacol.

[b0060] Brown S.S.G., Rutland J.W., Verma G., Feldman R.E., Alper J., Schneider M., Delman B.N., Murrough J.M., Balchandani P. (2019). Structural MRI at 7T reveals amygdala nuclei and hippocampal subfield volumetric association with Major Depressive Disorder symptom severity. Sci Rep.

[b0065] Chu S.-H., Lenglet C., Schreiner M.W., Klimes-Dougan B., Cullen K., Parhi K.K. (2018). Anatomical Biomarkers for Adolescent Major Depressive Disorder from Diffusion Weighted Imaging using SVM Classifier. Conf Proc IEEE Eng Med Biol Soc.

[b0070] Cîrstian R., Pilmeyer J., Bernas A., Jansen J.F.A., Breeuwer M., Aldenkamp A.P., Zinger S. (2023). Objective biomarkers of depression: A study of Granger causality and wavelet coherence in resting-state fMRI. J. Neuroimaging.

[b0075] Cole J., Costafreda S.G., McGuffin P., Fu C.H.Y. (2011). Hippocampal atrophy in first episode depression: a meta-analysis of magnetic resonance imaging studies. J Affect Disord.

[b0080] Dai L., Zhou H., Xu X., Zuo Z. (2019). Brain structural and functional changes in patients with major depressive disorder: a literature review. PeerJ.

[b0085] Desikan R.S., Ségonne F., Fischl B., Quinn B.T., Dickerson B.C., Blacker D., Buckner R.L., Dale A.M., Maguire R.P., Hyman B.T., Albert M.S., Killiany R.J. (2006). An automated labeling system for subdividing the human cerebral cortex on MRI scans into gyral based regions of interest. Neuroimage.

[b0090] Dichter G.S., Gibbs D., Smoski M.J. (2015). A systematic review of relations between resting-state functional-MRI and treatment response in major depressive disorder. J. Affect. Disord..

[b0095] Ding C., Peng H. (2005). Minimum redundancy feature selection from microarray gene expression data. J Bioinform Comput Biol.

[b0100] Drenthen G.S., Jansen J.F.A., Gommer E., Gupta L., Hofman P.A.M., van Kranen-Mastenbroek V.H., Hilkman D.M., Vlooswijk M.C.G., Rouhl R.P.W., Backes W.H. (2021). Predictive value of functional MRI and EEG in epilepsy diagnosis after a first seizure. Epilepsy Behav..

[b0105] Figee M., Riva-Posse P., Choi K.S., Bederson L., Mayberg H.S., Kopell B.H. (2022). Deep Brain Stimulation for Depression. Neurotherapeutics.

[b0110] Fischl B., Salat D.H., Busa E., Albert M., Dieterich M., Haselgrove C., van der Kouwe A., Killiany R., Kennedy D., Klaveness S., Montillo A., Makris N., Rosen B., Dale A.M. (2002). Whole Brain Segmentation: Automated Labeling of Neuroanatomical Structures in the Human Brain. Neuron.

[b0115] Fransson P., Marrelec G. (2008). The precuneus/posterior cingulate cortex plays a pivotal role in the default mode network: Evidence from a partial correlation network analysis. Neuroimage.

[b0120] Furukawa T.A., Akechi T., Azuma H., Okuyama T., Higuchi T. (2007). Evidence-Based Guidelines for Interpretation of the Hamilton Rating Scale for Depression. J. Clin. Psychopharmacol..

[b0125] Gautam S., Jain A., Gautam M., Vahia V.N., Grover S. (2017). Clinical Practice Guidelines for the management of Depression. Indian J Psychiatry.

[b0130] Grieve S.M., Korgaonkar M.S., Gordon E., Williams L.M., Rush A.J. (2016). Prediction of nonremission to antidepressant therapy using diffusion tensor imaging. J Clin Psychiatry.

[b0135] Hacimusalar Y., Eşel E. (2018). Suggested Biomarkers for Major Depressive Disorder. Noro Psikiyatr Ars.

[b0140] Hamilton M. (1960). A rating scale for depression. J Neurol Neurosurg Psychiatry.

[b0145] Hamilton J.P., Siemer M., Gotlib I.H. (2008). Amygdala volume in major depressive disorder: a meta-analysis of magnetic resonance imaging studies. Mol Psychiatry.

[b0150] Hamilton J.P., Farmer M., Fogelman P., Gotlib I.H. (2015). Depressive Rumination, the Default-Mode Network, and the Dark Matter of Clinical Neuroscience. Biol Psychiatry.

[b0155] Hariri A.R., Tessitore A., Mattay V.S., Fera F., Weinberger D.R. (2002). The Amygdala Response to Emotional Stimuli: A Comparison of Faces and Scenes. Neuroimage.

[b0160] Holtzheimer P.E., Mayberg H.S. (2011). Stuck in a rut: rethinking depression and its treatment. Trends Neurosci..

[b0165] Iglesias J.E., Augustinack J.C., Nguyen K., Player C.M., Player A., Wright M., Roy N., Frosch M.P., McKee A.C., Wald L.L., Fischl B., Van Leemput K. (2015). A computational atlas of the hippocampal formation using ex vivo, ultra-high resolution MRI: Application to adaptive segmentation of in vivo MRI. Neuroimage.

[b0170] Iglesias J.E., Van Leemput K., Augustinack J., Insausti R., Fischl B., Reuter M. (2016). Alzheimer’s Disease Neuroimaging Initiative, Bayesian longitudinal segmentation of hippocampal substructures in brain MRI using subject-specific atlases. Neuroimage.

[b0175] Jang J.H., Kim J.-H., Yun J.-Y., Choi S.-H., An S.C., Kang D.-H. (2018). Differences in Functional Connectivity of the Insula Between Brain Wave Vibration in Meditators and Non-meditators. Mindfulness (n y).

[b0180] Jenkinson M., Beckmann C.F., Behrens T.E., Woolrich M.W., Smith S.M. (2012). FSL. Neuroimage.

[b0185] Jeurissen B., Tournier J.-D., Dhollander T., Connelly A., Sijbers J. (2014). Multi-tissue constrained spherical deconvolution for improved analysis of multi-shell diffusion MRI data. Neuroimage.

[b0190] Jiang C., Yi L., Cai S., Zhang L. (2019). Ischemic Stroke in Pontine and Corona Radiata: Location Specific Impairment of Neural Network Investigated With Resting State fMRI. Front Neurol.

[b0195] Kang S.G., Cho S.E. (2020). Neuroimaging Biomarkers for Predicting Treatment Response and Recurrence of Major Depressive Disorder. Int. J. Mol. Sci..

[b0200] Kebets V., Favre P., Houenou J., Polosan M., Perroud N., Aubry J.-M., Van De Ville D., Piguet C. (2021). Fronto-limbic neural variability as a transdiagnostic correlate of emotion dysregulation. Transl Psychiatry.

[b0205] Korgaonkar M.S., Williams L.M., Song Y.J., Usherwood T., Grieve S.M. (2014). Diffusion tensor imaging predictors of treatment outcomes in major depressive disorder. Br. J. Psychiatry.

[b0210] Kraus C., Seiger R., Pfabigan D.M., Sladky R., Tik M., Paul K., Woletz M., Gryglewski G., Vanicek T., Komorowski A., Kasper S., Lamm C., Windischberger C., Lanzenberger R. (2019). Hippocampal Subfields in Acute and Remitted Depression-an Ultra-High Field Magnetic Resonance Imaging Study. Int J Neuropsychopharmacol.

[b0215] Kundu P., Voon V., Balchandani P., Lombardo M.V., Poser B.A., Bandettini P.A. (2017). Multi-echo fMRI: A review of applications in fMRI denoising and analysis of BOLD signals. NeuroImage, Cleaning up the fMRI Time Series: Mitigating Noise with Advanced Acquisition and Correction Strategies.

[b0220] Lacroix A., Calvet B., Laplace B., Lannaud M., Plansont B., Guignandon S., Balestrat P., Girard M. (2021). Predictors of clinical response after rTMS treatment of patients suffering from drug-resistant depression. Transl Psychiatry.

[b0225] Lai C.-H. (2019). Major depressive disorder in neuroimaging: What is beyond fronto-limbic model?. Current Psychiatry Research and Reviews.

[b0230] Lai C.-H. (2021). Fronto-limbic neuroimaging biomarkers for diagnosis and prediction of treatment responses in major depressive disorder. Prog. Neuropsychopharmacol. Biol. Psychiatry.

[b0235] Leaver A.M., Espinoza R., Joshi S.H., Vasavada M., Njau S., Woods R.P., Narr K.L. (2016). Desynchronization and Plasticity of Striato-frontal Connectivity in Major Depressive Disorder. Cereb Cortex.

[b0240] Lener M.S., Iosifescu D.V. (2015). In pursuit of neuroimaging biomarkers to guide treatment selection in major depressive disorder: a review of the literature. Ann. N. Y. Acad. Sci..

[b0245] Leucht S., Fennema H., Engel R., Kaspers–Janssen M., Lepping P., Szegedi A. (2013). What does the HAMD mean?. J. Affect. Disord..

[b0250] LeWinn K.Z., Connolly C.G., Wu J., Drahos M., Hoeft F., Ho T.C., Simmons A.N., Yang T.T. (2014). White matter correlates of adolescent depression: structural evidence for frontolimbic disconnectivity. J Am Acad Child Adolesc Psychiatry.

[b0255] Li T., Guo Y., Zhao Z., Chen M., Lin Q., Hu X., Yao Z., Hu B. (2024). Automated diagnosis of major depressive disorder with multi-modal MRIs based on contrastive learning: a few-shot study. IEEE Trans. Neural Syst. Rehabil. Eng..

[b0260] Ma H., Zhang D., Wang Y., Ding Y., Yang J., Li K. (2023). Prediction of early improvement of major depressive disorder to antidepressant medication in adolescents with radiomics analysis after ComBat harmonization based on multiscale structural MRI. BMC Psychiatry.

[b0265] Malhi G.S., Mann J.J. (2018). Depression. Lancet.

[b0270] Mangasarian, O.L., Bradley, P.S., 1998. Feature Selection Via Concave Minimization and Support Vector Machines.

[b0275] Mann H.B., Whitney D.R. (1947). On a Test of Whether one of Two Random Variables is Stochastically Larger than the Other. Ann. Math. Stat..

[b0280] McKinnon M.C., Yucel K., Nazarov A., MacQueen G.M. (2009). A meta-analysis examining clinical predictors of hippocampal volume in patients with major depressive disorder. J Psychiatry Neurosci.

[b0285] Mueller S., Costa A., Keeser D., Pogarell O., Berman A., Coates U., Reiser M.F., Riedel M., Möller H., Ettinger U., Meindl T. (2014). The effects of methylphenidate on whole brain intrinsic functional connectivity. Hum Brain Mapp.

[b0290] Pei C., Sun Y., Zhu J., Wang X., Zhang Y., Zhang S., Yao Z., Lu Q. (2020). Ensemble Learning for Early-Response Prediction of Antidepressant Treatment in Major Depressive Disorder. J. Magn. Reson. Imaging.

[b0295] Perlman K., Benrimoh D., Israel S., Rollins C., Brown E., Tunteng J.-F., You R., You E., Tanguay-Sela M., Snook E., Miresco M., Berlim M.T. (2019). A systematic meta-review of predictors of antidepressant treatment outcome in major depressive disorder. J Affect Disord.

[b0300] Pilmeyer J., Huijbers W., Lamerichs R., Jansen J.F.A., Breeuwer M., Zinger S. (2022). Functional MRI in major depressive disorder: A review of findings, limitations, and future prospects. J. Neuroimaging.

[b0305] Pilmeyer J., Hadjigeorgiou G., Lamerichs R.M.J.N., Breeuwer M., Aldenkamp A.P., Zinger S. (2023). Spatial and Temporal Quality of Brain Networks for Different Multi-Echo fMRI Combination Methods. IEEE Access.

[b0310] Poirot M.G., Ruhe H.G., Mutsaerts H.-J.-M.-M., Maximov I.I., Groote I.R., Bjørnerud A., Marquering H.A., Reneman L., Caan M.W.A. (2024). Treatment Response Prediction in Major Depressive Disorder Using Multimodal MRI and Clinical Data: Secondary Analysis of a Randomized Clinical Trial. AJP.

[b0315] Posse S., Wiese S., Gembris D., Mathiak K., Kessler C., Grosse-Ruyken M.-L., Elghahwagi B., Richards T., Dager S.R., Kiselev V.G. (1999). Enhancement of BOLD-contrast sensitivity by single-shot multi-echo functional MR imaging. Magn. Reson. Med..

[b0320] Ruhé H.G., Dekker J.J., Peen J., Holman R., de Jonghe F. (2005). Clinical use of the Hamilton Depression Rating Scale: is increased efficiency possible? A post hoc comparison of Hamilton Depression Rating Scale, Maier and Bech subscales, Clinical Global Impression, and Symptom Checklist-90 scores. Compr. Psychiatry.

[b0325] Samuels B.A., Leonardo E.D., Hen R. (2015). Hippocampal Subfields and Major Depressive Disorder. Biol Psychiatry.

[b0330] Sanjuan P.M., Thoma R., Claus E.D., Mays N., Caprihan A. (2013). Reduced white matter integrity in the cingulum and anterior corona radiata in posttraumatic stress disorder in male combat veterans: A diffusion tensor imaging study. Psychiatry Res.

[b0335] Saygin Z.M., Kliemann D., Iglesias J.E., van der Kouwe A.J.W., Boyd E., Reuter M., Stevens A., Van Leemput K., McKee A., Frosch M.P., Fischl B., Augustinack J.C. (2017). Alzheimer’s Disease Neuroimaging Initiative, High-resolution magnetic resonance imaging reveals nuclei of the human amygdala: manual segmentation to automatic atlas. Neuroimage.

[b0340] Schmaal L., Marquand A.F., Rhebergen D., van Tol M.-J., Ruhé H.G., van der Wee N.J.A., Veltman D.J., Penninx B.W.J.H. (2015). Predicting the Naturalistic Course of Major Depressive Disorder Using Clinical and Multimodal Neuroimaging Information: A Multivariate Pattern Recognition Study. Biological Psychiatry, Depression.

[b0345] Shapiro S.S., Wilk M.B. (1965). An Analysis of Variance Test for Normality (Complete Samples). Biometrika.

[b0350] Shi, Q., Chen, H., Jia, Q., Yuan, Z., Wang, J., Li, Y., Han, Z., Mo, D., Zhang, Y., 2020. Altered Granger Causal Connectivity of Resting-State Neural Networks in Patients With Leukoaraiosis-Associated Cognitive Impairment—A Cross-Sectional Study. Frontiers in Neurology 11. 10.3389/fneur.2020.00457 PMC7325959 32655471

[b0355] Smith, R.E., Raffelt, D., Tournier, J.-D., Connelly, A., 2022. Quantitative streamlines tractography: methods and inter-subject normalisation. Aperture Neuro 1–25. 10.52294/ApertureNeuro.2022.2.NEOD9565.

[b0360] Smith S.M., Fox P.T., Miller K.L., Glahn D.C., Fox P.M., Mackay C.E., Filippini N., Watkins K.E., Toro R., Laird A.R., Beckmann C.F. (2009). Correspondence of the brain’s functional architecture during activation and rest. PNAS.

[b0365] Smith R.E., Tournier J.-D., Calamante F., Connelly A. (2012). Anatomically-constrained tractography: Improved diffusion MRI streamlines tractography through effective use of anatomical information. Neuroimage.

[b0370] Smith R.E., Tournier J.-D., Calamante F., Connelly A. (2015). SIFT2: Enabling dense quantitative assessment of brain white matter connectivity using streamlines tractography. Neuroimage.

[b0375] Sorg C., Manoliu A., Neufang S., Myers N., Peters H., Schwerthöffer D., Scherr M., Mühlau M., Zimmer C., Drzezga A., Förstl H., Bäuml J., Eichele T., Wohlschläger A.M., Riedl V. (2013). Increased Intrinsic Brain Activity in the Striatum Reflects Symptom Dimensions in Schizophrenia. Schizophr. Bull..

[b0380] Stuhrmann A., Suslow T., Dannlowski U. (2011). Facial emotion processing in major depression: a systematic review of neuroimaging findings. Biol Mood Anxiety Disord.

[b0385] Tournier J.-D., Smith R., Raffelt D., Tabbara R., Dhollander T., Pietsch M., Christiaens D., Jeurissen B., Yeh C.-H., Connelly A. (2019). MRtrix3: A fast, flexible and open software framework for medical image processing and visualisation. Neuroimage.

[b0390] van Velzen L.S., Kelly S., Isaev D., Aleman A., Aftanas L.I., Bauer J., Baune B.T., Brak I.V., Carballedo A., Connolly C.G., Couvy-Duchesne B., Cullen K.R., Danilenko K.V., Dannlowski U., Enneking V., Filimonova E., Forster K., Frodl T., Gotlib I.H., Groenewold N.A., Grotegerd D., Harris M.A., Hatton S.N., Hawkins E.L., Hickie I.B., Ho T.C., Jansen A., Kircher T., Klimes-Dougan B., Kochunov P., Krug A., Lagopoulos J., Lee R., Lett T.A., Li M., MacMaster F.P., Martin N.G., McIntosh A.M., McLellan Q., Meinert S., Nenadic I., Osipov E., Penninx B.W.J.H., Portella M.J., Repple J., Roos A., Sacchet M.D., Samann P.G., Schnell K., Shen X., Sim K., Stein D.J., van Tol M.-J., Tomyshev A.S., Tozzi L., Veer I.M., Vermeiren R., Vives-Gilabert Y., Walter H., Walter M., van der Wee N.J.A., van der Werff S.J.A., Schreiner M.W., Whalley H.C., Wright M.J., Yang T.T., Zhu A., Veltman D.J., Thompson P.M., Jahanshad N., Schmaal L. (2019). White matter disturbances in major depressive disorder: a coordinated analysis across 20 international cohorts in the ENIGMA MDD working group. Mol Psychiatry.

[b0395] van Vliet I.M., de Beurs E. (2007). The MINI-International Neuropsychiatric Interview. A brief structured diagnostic psychiatric interview for DSM-IV en ICD-10 psychiatric disorders. Tijdschr Psychiatr.

[b0400] Varol E., Gaonkar B., Erus G., Schultz R., Davatzikos C. (2012). IEEE International Symposium on Biomedical Imaging (ISBI). Presented at the 2012 9th IEEE International Symposium on Biomedical Imaging (ISBI).

[b0405] Wang X., Foryt P., Ochs R., Chung J.-H., Wu Y., Parrish T., Ragin A.B. (2011). Abnormalities in Resting-State Functional Connectivity in Early Human Immunodeficiency Virus Infection. Brain Connect.

[b0410] Yang C., Wang P., Tan J., Liu Q., Li X. (2021). Autism spectrum disorder diagnosis using graph attention network based on spatial-constrained sparse functional brain networks. Comput. Biol. Med..

[b0415] Zhang S., She S., Qiu Y., Li Z., Wu X., Hu H., Zheng W., Huang R., Wu H. (2023). Multi-modal MRI measures reveal sensory abnormalities in major depressive disorder patients: A surface-based study. NeuroImage: Clinical.

[b0420] Zhou H.-X., Chen X., Shen Y.-Q., Li L., Chen N.-X., Zhu Z.-C., Castellanos F.X., Yan C.-G. (2020). Rumination and the default mode network: Meta-analysis of brain imaging studies and implications for depression. Neuroimage.

