# Supplementary material for: Multi-modal MRI for objective diagnosis and outcome prediction in depression
Source: Neuroimage Clin. 2024 Oct 10;44:103682. doi: 10.1016/j.nicl.2024.103682 (PMC11663772; doi:10.1016/j.nicl.2024.103682)
Supplement: Supplementary Data 1 [file mmc1.docx]

Supplementary Material

Multi-modal MRI for objective diagnosis and outcome prediction in depression

**Jesper Pilmeyer^*^, Rolf Lamerichs, Sjir Schielen, Faroeq Ramsaransing, Vivianne van Kranen-Mastenbroek, Jacobus F.A. Jansen, Marcel Breeuwer, Svitlana Zinger**

*** Correspondence:** Jesper Pilmeyer: [j.pilmeyer@tue.nl](mailto:j.pilmeyer@tue.nl)

# Supplementary Information

## MRI acquisition details

# All participants underwent MRI scanning at Expertise Centre For Epilepsy and Sleep Disorders Kempenhaeghe (Heeze, the Netherlands) using a Philips Achieva dStream 3T scanner (Philips Healthcare, Best, the Netherlands).

# The anatomical T_1_-weighted images were acquired with a 3D turbo field echo sequence with the following parameters: repetition time (TR) = 8.1 ms, echo time (TE) = 3.7 ms, 1mm isotropic voxel resolution (256 x 256 x 180 matrix), flip angle = 8°, compressed SENSE accelerating factor = 4.6.

# The anatomical T_2_-weighted sequence was a coronal-oblique turbo spin-echo with TR = 4500, TE = 85 ms, a 0.39 x 0.39 x 2 mm voxel resolution (512 x 512 x 30 matrix) and EPI factor = 1. The field-of-view was set such that it completely included the hippocampi and amygdalae.

# The fMRI images were acquired using an MBME echo-planar imaging sequence with a 2.29 x 2.29 x 2.70 mm3 voxel resolution (96 x 96 x 51 matrix), 380 volumes, TR = 1350 ms, number of echoes = 3 at TE = 11.3, 31.8, 52.3 ms, flip angle = 73°, multiband factor 3, SENSE accelerating factor 2.5. During MRI acquisition, a photoplethysmographic unit was placed on a finger and a respiratory belt was placed on the abdomen to externally measure cardiac and respiratory signals, respectively.

# For the diffusion-weighted images, a single-shot, multiband, spin-echo EPI sequence was used with a 1.67 x 1.67 x 1.7 mm voxel resolution (144 x 144 x 81 matrix), TR = 5300 ms, TE = 96 ms, 102 volumes, 4 diffusion-weighted shells (7, 6, 15, 15 and 59 volumes for the 0, 500, 1000, 2000 and 3000 s/mm2 diffusion gradients, respectively), a total of 96 diffusion directions, a posterior-anterior phase-encoding direction, flip angle = 78°, EPI factor = 141, multiband factor = 3. Additionally, a pair of b = 0 s/mm2 fieldmap scans with identical and reversed (anterior-posterior) phase-encoding directions was acquired to correct for EPI susceptibility distortions.

## MRI processing

T_1_-weighted brain volumes and cortical thickness measures were obtained using Freesurfer’s recon-all (<https://surfer.nmr.mgh.harvard.edu/fswiki/recon-all>), version 7.1.1. Volumes of hippocampal subfields and amygdalar nuclei were extracted using both, the high resolution T_2_-weighted scan and T_1_-weighted scan, following Freesurfer’s segmentHA_T2 script (with the additional argument USE_T1 set to True) (Iglesias et al., 2016, 2015; Saygin et al., 2017).

fMRI preprocessing was performed using Statistical Parametric Mapping software (SPM12, <https://www.fil.ion.ucl.ac.uk/spm/>, RRID:SCR_007037) in Matlab R2022b (The MathWorks Inc, Natick, Massachusetts, RRID:SCR_001622) and additional functions from the FMRIB Software Library v6.0 (FSL, RRID:SCR_002823) package (Jenkinson et al., 2012). First, minimal preprocessing was performed before echo time-series combination, as described previously (DuPre et al., 2021), including slice timing correction on each separate echo time-series (SPM12), realignment transformation (6 degrees-of-freedom) estimation on the echo 2 (TE = 31.8 ms) time-series using the first volume as reference image (FSL’s MCFLIRT function) and applying these estimated realignment parameters to all echo time-series (FSL’s FLIRT function). Subsequently, the three echo time-series were combined based on the ‘optimal combination’ algorithm (Posse et al., 1999) to increase temporal signal-to-noise ratio, reduce signal loss from susceptibility artifacts and improve the resting-state network quality (Kundu et al., 2017; Pilmeyer et al., 2023). Using SPM12, the T_1_-weighted scan was coregistered to the fMRI scan and segmented into six separate tissue classes (including white and gray matter, cerebral spinal fluid). During the segmentation process, linear 12 degrees-of-freedom transformation matrices for spatial normalization to Montreal Neurological Institute space were estimated. Spatial normalization was performed by applying these transformation matrices to the fMRI images. Confound signal regression of the estimated six motion regressors, their first order derivatives and the first 5 principal components of the white matter (WM) and cerebral spinal fluid was performed through aComPOr, implemented in the CONN toolbox (Behzadi et al., 2007; Whitfield-Gabrieli and Nieto-Castanon, 2012). A conservative Butterworth bandpass filter with 0.01 and 0.2 Hz cutoffs (Pilmeyer et al., 2023) and spatial smoothing with a 5 mm full-width at half-maximum kernel were applied in SPM12.

Functional resting-state brain networks (RSNs) were extracted using group independent component analysis (ICA) with the Group ICA of fMRI Toolbox (GIFT v3.0c, <http://icatb.sourceforge.net/>, RRID:SCR_001953) and the Infomax algorithm (Bell and Sejnowski, 1995). This resulted in 35 spatially independent components (ICs). A number of 35 ICs was found to be the optimal number of components to not split RSNs into subnetworks or obtain merged RSNs within a single component (Wang et al., 2011). Individual spatial maps and time-series were derived using group ICA back-reconstruction (Calhoun et al., 2001). The time-series were converted to z-scores. A total number of 15 RSNs were identified from the 35 ICs based on a goodness-of-fit approach (Bernas et al., 2018) between ICs and the RSN atlas of Smith et al. (Smith et al., 2009). Visual inspection was used to verify the automated identification process. These included the primary visual network (pVN), lateral visual network (latVN), occipital visual network (oVN), default mode network (DMN), cerebellum network (CN), sensorimotor network (SMN), auditory network (AN) and left and right frontoparietal network (l/rFPN). The DMN was further separated into the whole, anterior and posterior components (DMN, aDMN and pDMN), and the SMN also contained a lateral SMN (latSMN) component. Visual inspection identified 3 additional networks that are not part of the Smith et al. atlas: the salience network (SN), basal ganglia network (BN) and dorsal attention network (DAN) were identified based on previous RSN studies (Bharti et al., 2022; Mueller et al., 2014; Shi et al., 2020; Sorg et al., 2013). Thus, in total 15 RSNs were found as shown in Figure S2.

DTI images were processed using MRtrix3 (Tournier et al., 2019) and FSL (Jenkinson et al., 2012) which included denoising based on Marchenko-Pastur principal component analysis (dwidenoise), motion and eddy current-induced distortion correction (dwifslpreproc) and susceptibility-induced distortion correction with an anterior-posterior and posterior-anterior phase-encoding fieldmap pair (topup). Then, bias correction was applied (dwibiascorrect), followed by removal of non-brain tissue (dwi2mask). Structural connectivity was obtained by deriving fiber orientation distribution maps with multi-shell, multi-tissue constrained spherical deconvolution (CSD) (Jeurissen et al., 2014) using the dhollander algorithm (estimating gray matter (GM), WM, cerebral spinal fluid response function (55–58)) (Dhollander et al., 2019). Subsequently, a GM-WM boundary mask was generated (5ttgen) (Smith et al., 2012), followed by registration of the mean pre-processed b=0 scan to the GM-WM mask (FLIRT) (Jenkinson and Smith, 2001) and back transformation to the original subject space. Anatomically constrained tractography (ACT) was applied to ensure streamlines were anatomically and biologically plausible (Smith et al., 2012). The maximum streamline length was 250mm and the minimum fractional anisotropy cutoff 0.06. A total of 20 million streamlines was generated after which weights were assigned to each streamline (SIFT2) (Smith et al., 2015). These weights are based on minimizing the difference between the streamline density and the estimated fiber density from the diffusion model to reflect the underlying biological connectivity (Smith et al., 2022).

## Feature extraction

### T_1_-weighted

From the T_1_-weighted images, volumes of 16 subcortical structures (Fischl et al., 2002) and the cortical thickness of 68 cortical structures were obtained (Desikan et al., 2006). Finally, Freesurfer’s recon-all function extracts volumes of 29 other brain regions, including the brain stem, ventral diencephalon (includes the hypothalamus and substantia nigra) and white matter hyperintensities. Thus, a total of 113 elements were obtained for the T_1_-based feature set.

### T_2_-weighted

The volumes of 44 hippocampal subfields and 20 amygdalar nuclei were extracted from the high-resolution T_2_-weighted scans in combination with the T_1_-weighted scan, resulting in a total of 66 elements. These were calculated based on Freesurfer’s hippocampus subfields and amygdalae nuclei atlases (Iglesias et al., 2016, 2015; Saygin et al., 2017).

### DTI

Five measures of structural connectivity matrices were obtained between 84 subcortical and cortical regions (similar to the extracted T_1_-weighted regions: from the default atlas in Freesurfer (Desikan et al., 2006; Fischl et al., 2002)) with the tck2connectome tool (Tournier et al., 2019). Mean diffusivity (MD), FA, axial diffusivity (AD) and radial diffusivity (RD) were obtained along multiple points of each streamline and then averaged to obtain the average over each streamline. The value in each cell in the 84 x 84 matrix represents the average measure of structural connectivity as calculated over all streamlines that connect two regions-of-interest (ROIs), scaled by the streamline weights as described before. In addition, the sum of streamline weights (sSW) was obtained. This measure is a more biologically accurate measure of white matter density of connections between ROIs (Smith et al., 2015). Likewise, a 84 x 84 matrix was obtained for the sSW but the values were scaled by the inverse of the volumes of the ROI pair (Merenstein et al., 2023). Only the upper matrix was used as final feature set, resulting in 84 x (84-1) / 2 = 3486 elements for each of the 5 DTI feature sets.

### Task-based fMRI

A design matrix was created to obtain activation contrast maps between conditions. The design matrix included regressors of the 3 conditions (rest, shapes, faces), and 25 physiologically-derived regressors. The latter include the externally measured cardiac and respiratory signals after modeling by the RETROICOR software tool (Glover et al., 2000). In total, the 25 physiologically-derived regressors comprised 6 cardiac, 8 respiratory, 4 multiplicative, 1 respiration-volume-per time x respiratory response function and 6 motion regressors. The t-value activity contrast maps were calculated between the Faces > Shapes and Faces > Rest conditions in 11 ROIs. These ROIs were chosen based on previous studies that found either abnormal activity in these regions during the Hariri task in MDD participants or because they were associated with emotion regulation by acting directly on the amygdala, the primary activation target in this paradigm (Hariri et al., 2000; Townsend et al., 2010). A total of 11 ROIs for 2 contrasts of interest resulted in 22 elements in the activation contrast feature set. These comprised bilateral hippocampi, amygdalae, and bilateral parahippocampal and fusiform gyri. Moreover, the ventromedial prefrontal cortex, anterior cingulate cortex (ACC) and subgenual were extracted. For more details, see our previous paper (Pilmeyer et al., 2024)

### Resting-state fMRI

FC matrices of the 15 x 15 RSNs were created by calculating the Pearson correlation between the time-series, followed by z-scoring. Since FC is a static approach, we also evaluated the value of a neurodynamic approach. For this, wavelet coherence analysis (WCA) was performed, which assesses the temporally changing interactions between RSNs. Two features were derived from WCA: lead coherence (leadCoh) and the number of coherence clusters (nCC) as described by Cîrstian et al. using the same parameters (Cîrstian et al., 2023). However, a total of 50 periods were obtained for the analysis and bins with periods < 5 s (> 0.2 Hz) were discarded because of the previously applied bandpass filter. LeadCoh reflects a causal effect between pairs of RSNs, i.e. RSN 1 leads in phase compared to RSN 2. nCC describes the amount of different interactions (clusters) that occur between RSN pairs during the resting-state fMRI scan, including the time-series being in-phase, out-of-phase, leading or lagging. For FC and nCC, the upper matrix was taken to remove duplicate elements of the feature vector, i.e. 15 x (15-1) / 2 = 105 elements. For leadCoh, the matrix is not symmetrical, meaning that a 15 x 14 = 210 element was used.

# Supplementary Figures


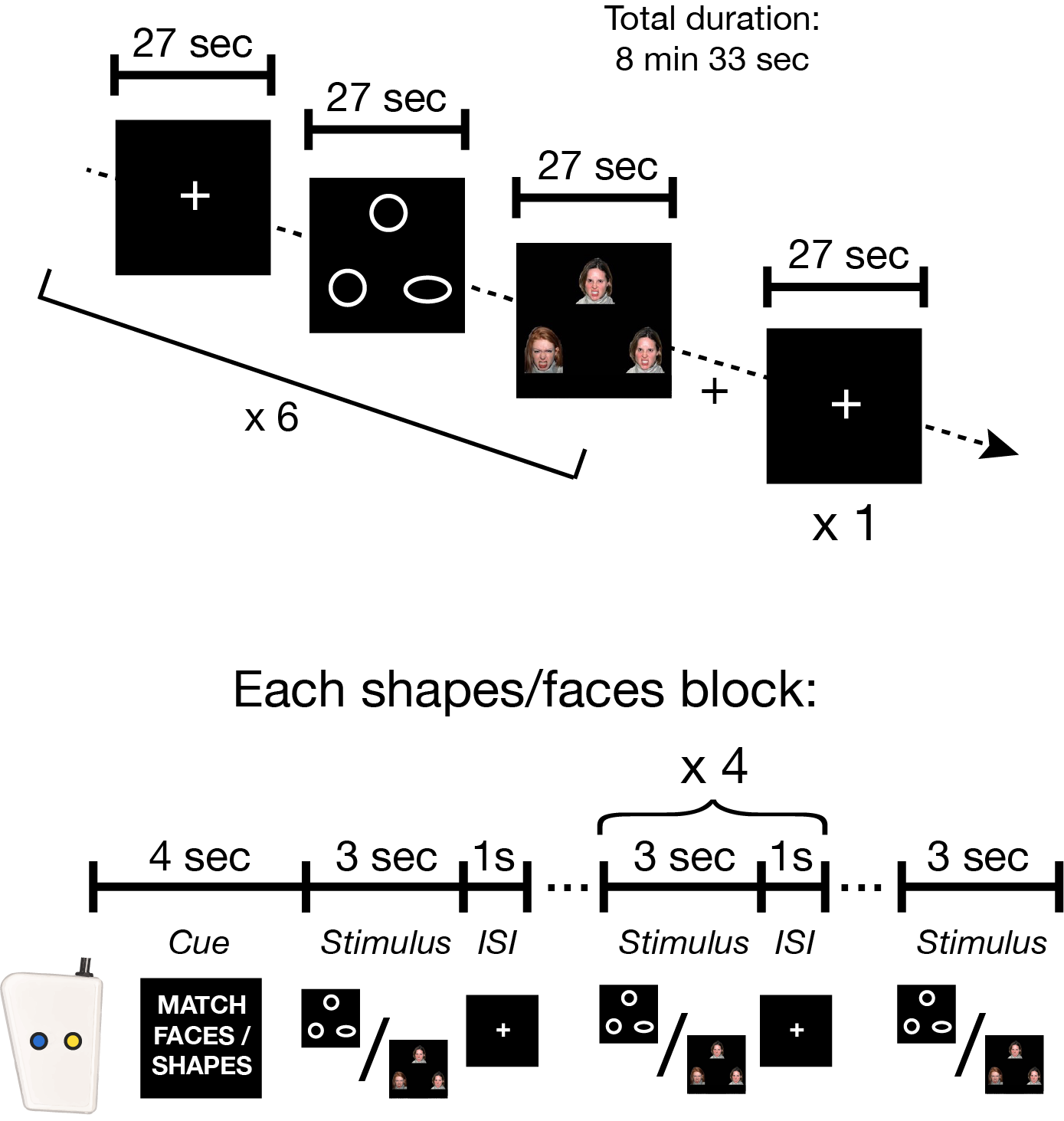


**Supplementary Figure S1** The Hariri paradigm is a face-matching task, aimed at stimulating brain areas that are associated with processing of negative emotions from faces. It contains 7 blocks of rest (cross), 6 periods of matching shapes and 6 period of matching negative emotions (anger and fear). Participants are instructed to use the button press (shown in the image left to the cue block) to match the left or right lower image to the upper image during the shapes and faces blocks. Each block lasts 27 seconds and contains a cue (4 sec) with instructions, 6 stimuli (3 sec each) and an inter-stimulus interval (ISI) of 1 sec between each stimulus. Blocks are interchanged: it starts with a rest block, followed by shapes and faces blocks This procedure is repeated 6 times and ends with an additional rest block. Abbreviations: sec/s = seconds; ISI = inter-stimulus interval.

*
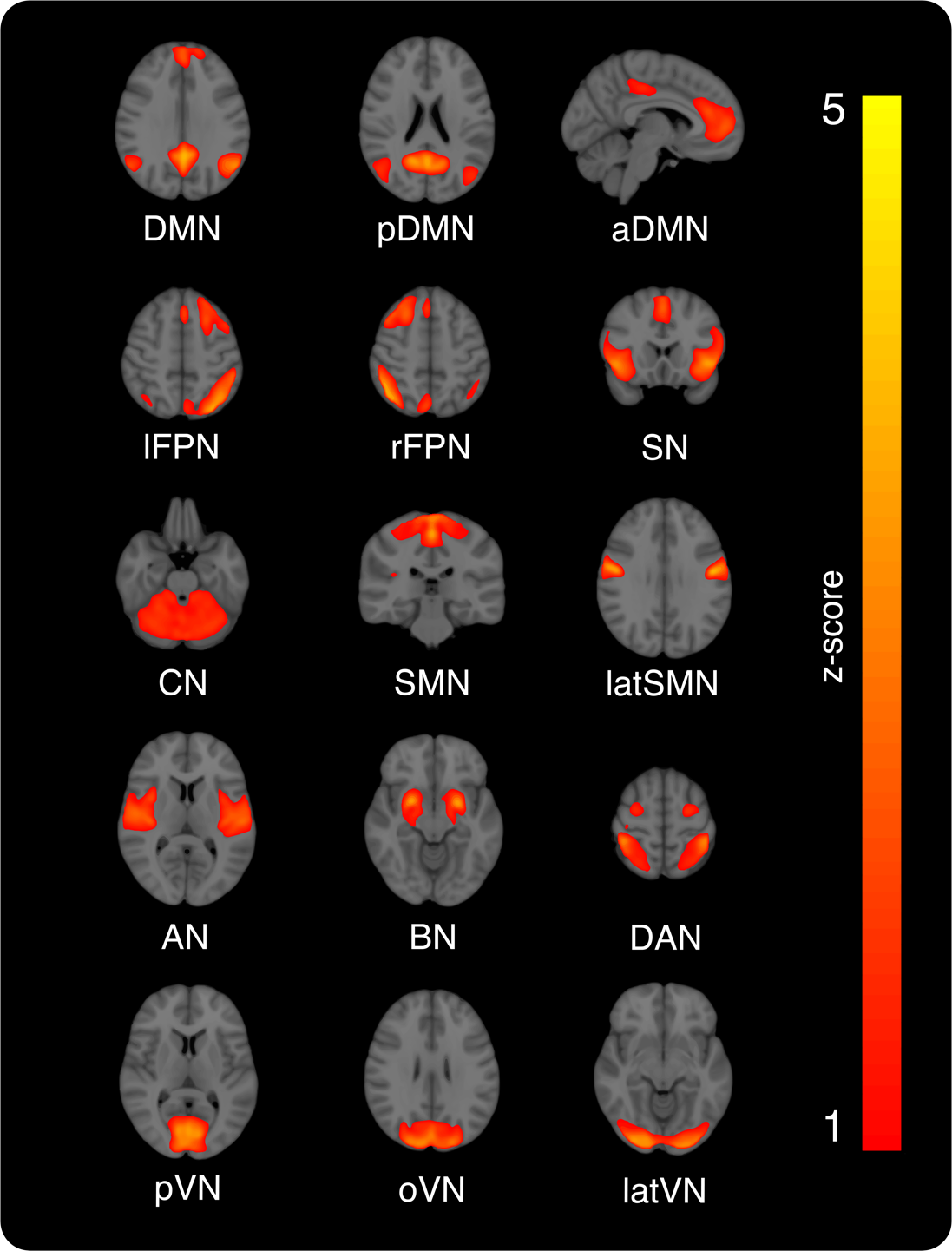
*

**Supplementary Figure S2** The 15 identified group resting-state networks in this study obtained via independent component analysis. The colors represent the z-score. Abbreviations: default mode network (DMN); posterior/anterior DMN (pDMN/aDMN); left/right frontoparietal network (l/rFPN); salience network (SN); cerebellum network (CN); sensorimotor network (SMN); lateral SMN (latSMN); auditory network (AN); basal ganglia network (BN); dorsal attention network (DAN); primary/occipital/lateral visual network (p/o/latVN).

## Supplementary Tables

| **Uni-modal feature sets information** | | | | | | | | |  |
| --- | --- | --- | --- | --- | --- | --- | --- | --- | --- |
| **Feature set** | **Modality** | **n feature**  **elements** |  | **k_initial_** | **k_final_** | **k_optimal_**  **(diagnosis)** | **k_optimal_**  **(outcome**  **prediction)** | **Description** | |
| T_1_ | T_1_ | 113 |  | 10 | 5 | 2.97 ± 1.51 | 2.87 ± 1.34 | Volumes and cortical thickness | |
| T_2_ | T_2_ | 66 |  | 10 | 5 | 3.40 ± 1.39 | 2.71 ± 1.47 | Volumes of hippocampal subfields and amygdalar nuclei | |
| sFC | Resting-state fMRI | 105 |  | 10 | 5 | 2.83 ± 1.49 | 2.97 ± 1.28 | Static functional connectivity: Z-transformed Pearson correlation between RSN time-series | |
| nCC | Resting-state fMRI | 105 |  | 10 | 5 | 3.08 ± 1.41 | 2.68 ± 1.35 | Number of coherence clusters in WCA map, reflecting the total amount and duration of RSN interactions | |
| leadCoh | Resting-state fMRI | 210 |  | 10 | 5 | 3.06 ± 1.50 | 2.84 ± 1.53 | Lead coherence, the averaged time of RSN 1 leading RSN 2 (by phase), reflecting a time-varying causal relationship | |
| Act | Task-based fMRI | 22 |  | 10 | 5 | 2.37 ± 1.37 | 2.68 ± 1.51 | Activity contrast between faces-rest and faces-shapes condition in an emotional face-matching task, reflecting associated brain activity between both contrasts. | |
| sSW | DTI | 3486 |  | 10 | 5 | 2.75 ± 1.54 | 2.48 ± 1.59 | Sum of streamline weights between two regions, reflecting a biologically accurate measure of white matter density / connectivity | |
| MD | DTI | 3486 |  | 10 | 5 | 2.89 ± 1.55 | 3.10 ± 1.42 | Mean diffusivity, reflecting membrane density | |
| FA | DTI | 3486 |  | 10 | 5 | 2.98 ± 1.59 | 3.13 ± 1.52 | Fractional anisotropy, a measure of white matter integrity | |
| AD | DTI | 3486 |  | 10 | 5 | 3.16 ± 1.54 | 3.26 ± 1.55 | Axial diffusivity, a measure associated with axonal properties | |
| RD | DTI | 3486 |  | 10 | 5 | 3.11 ± 1.43 | 2.97 ± 1.37 | Radial diffusivity, reflecting myelin properties | |

**Supplementary Table S1** The different feature sets that are used for classification. The rows show the modalities from which they were extracted, the number of feature elements, the number of feature elements after initial feature selection (k_initial_), the number of feature elements after the second round of feature selection based on ranking (k_final_), the optimal number of feature elements that yielded the highest performance metrics (k_optimal_) and a description of each feature set. Abbreviations: : sFC = static functional connectivity; nCC = number of coherence clusters; leadCoh = lead coherence; Act = activity; sSW = sum of streamline weights; MD = mean diffusivity; FA = fractional anisotropy; AD = axial diffusivity; RD = radial diffusivity; fMRI = functional MRI; DTI = diffusion tensor imaging; RSN = resting-state network; WCA = wavelet coherence analysis.

|  | **Uni-modal test performance - Diagnosis** | | | | | | | | |  |
| --- | --- | --- | --- | --- | --- | --- | --- | --- | --- | --- |
| **Feature set** | **Feature selection** | **AUC** | | **Acc [%]** | **Sens [%]** | **Spec [%]** | **F1-score** | **Prec** | **k_optimal_** | **p-value** |
| T_1_ | MRMR | 0.672  [0.671, 0.678] | | 61.9  [61.8, 62.5] | **71.9**  **[71.6, 72.5]** | 51.6  [51.3, 52.5] | 0.657  [0.651, 0.660] | 0.605  [0.601, 0.611] | 2.97  (± 1.51) | <0.05 |
| T_2_ | CV-SVM | 0.621  [0.617, 0.625] | | 55.6  [55.1, 55.9] | 68.8  [68.3, 69.4] | 41.9  [41.2, 42.3] | 0.611  [0.603, 0.612] | 0.550  [ 0.544, 0.554] | 3.40  (± 1.39) | n.s. |
| sFC | CV-SVM | 0.558  [0.553, 0.561] | | 55.6  [55.0, 55.8] | 56.3  [54.1, 56.7] | 54.8  [54.1, 55.3] | 0.563  [0.555, 0.564] | 0.563  [0.560, 0.571] | 2.83  (± 1.49) | n.s. |
| nCC | MRMR | 0.632  [0.632, 0.640] | | 60.3  [60.4, 61.2] | 65.6  [65.6, 66.6] | 54.8  [54.7, 55.8] | 0.627  [0.624, 0.633] | 0.600  [0.600, 0.610] | 3.08  (± 1.41) | <0.05 |
| leadCoh | MRMR | 0.588  [0.582 0.590] | | 54.0  [53.4, 54.2] | 43.8  [43.0, 44.0] | 64.5  [63.9, 65.0] | 0.491  [0.478, 0.488] | 0.560  [0.549, 0.562] | 3.06  (± 1.50) | n.s. |
| Act | MRMR | 0.415  [0.410, 0.418] | | 46.0  [45.6, 46.4] | 46.9  [46.1, 47.2] | 45.2  [44.7, 45.8] | 0.469  [0.459, 0.469] | 0.469  [0.463, 0.474] | 2.37  (± 1.37) | n.s. |
| sSW | MRMR | 0.539  [0.535, 0.543] | | 47.6  [47.2, 48.0] | 53.1  [52.8, 53.9] | 41.9  [41.3, 42.4] | 0.507  [0.499, 0.508] | 0.486  [0.478, 0.488] | 2.75  (± 1.54) | n.s. |
| MD | CV-SVM | **0.701**  **[0.699 0.706]** | | **69.8**  **[69.6, 70.3]** | **71.9**  **[71.7, 72.7]** | **67.7**  **[67.3, 68.3]** | **0.708**  **[0.703, 0.711]** | **0.697**  **[0.694, 0.704]** | 2.89  (± 1.55) | <0.01 |
| FA | CV-SVM | 0.530  [0.528, 0.535] | | 54.0  [53.7, 54.5] | 40.6  [40.3, 41.3] | **67.7**  **[67.3, 68.4]** | 0.473  [0.466, 0.476] | 0.565  [0.563, 0.576] | 2.98  (± 1.59) | n.s. |
| AD | MRMR | 0.475  [0.472, 0.480] | | 50.8  [50.5, 51.3] | 37.5  [36.7, 37.7] | 64.5  [64.4, 65.6] | 0.436  [0.424, 0.434] | 0.522  [0.514, 0.527] | 3.16  (± 1.54) | n.s. |
| RD | CV-SVM | 0.469  [0.467, 0.475] | | 49.2  [49.0, 49.8] | 37.5  [37.2, 38.3] | 61.3  [60.9, 62.0] | 0.429  [0.421, 0.432] | 0.500  [0.496, 0.509] | 3.11  (± 1.43) | n.s. |
|  | **Uni-modal test performance – Outcome prediction** | | | | | | | | |  |
| **Feature set** | **Feature selection** | | **AUC** | **Acc [%]** | **Sens [%]** | **Spec [%]** | **F1-score** | **Prec** | **k_optimal_** | **p-value** |
| T_1_ | MRMR | | 0.680  [0.677, 0.688] | 64.5  [63.9, 65.0] | 58.3  [58.4, 60.3] | 68.4  [67.2, 68.6] | 0.560  [0.551, 0.566] | 0.538  [0.538, 0.555] | 2.87  (± 1.34) | n.s. |
| T_2_ | CV-SVM | | 0.303  [0.296, 0.306] | 48.4  [47.7, 48.8] | 25.0  [24.1, 25.7] | 63.2  [62.2, 63.6] | 0.273  [0.264, 0.278] | 0.300  [0.285, 0.303] | 2.71  (± 1.47) | n.s. |
| sFC | MRMR | | 0.553  [0.548, 0.560] | 51.6  [51.1, 52.2] | 58.3  [57.8, 59.6] | 47.4  [46.5, 47.9] | 0.483  [0.469, 0.483] | 0.412  [0.405, 0.421] | 2.97  (± 1.28) | n.s. |
| nCC | CV-SVM | | 0.772  [0.768, 0.778] | **74.2**  **[73.7, 74.7]** | 66.7  [66.0, 67.7] | 78.9  [78.3, 79.5] | 0.667  [0.653, 0.667] | 0.667  [0.662, 0.679] | 2.68  (± 1.35) | <0.01 |
| leadCoh | MRMR | | 0.614  [0.610, 0.621] | 71.0  [70.6, 71.6] | 58.3  [57.7, 59.5] | 78.9  [78.5, 79.7] | 0.609  [0.592, 0.607] | 0.636  [0.629, 0.648] | 2.84  (± 1.53) | n.s. |
| Act | MRMR | | 0.544  [0.536, 0.548] | 51.6  [50.8, 51.9] | 50.0  [49.4, 51.2 ] | 52.6  [51.2, 52.6] | 0.444  [0.427, 0.442] | 0.400  [0.389, 0.406] | 2.68  (± 1.51) | n.s. |
| sSW | CV-SVM | | **0.860**  **[0.858, 0.866]** | **77.4**  **[77.0, 77.9]** | **91.7**  **[91.3, 92.3]** | 68.4  [67.9, 69.3] | **0.759**  **[0.746, 0.758]** | 0.647  [0.639, 0.654] | 2.48  (± 1.59) | <0.001 |
| MD | CV-SVM | | 0.636  [0.634, 0.645] | 58.1  [57.0, 59.0] | 66.7  [65.7, 67.5] | 52.6  [52.6, 54.1] | 0.552  [0.535, 0.550] | 0.471  [0.463, 0.478] | 3.10  (± 1.42) | n.s. |
| FA | MRMR | | 0.645  [0.642, 0.652] | 71.0  [70.8, 71.8] | 41.7  [41.3, 43.1] | **89.5**  **[88.9, 89.8]** | 0.526  [0.508, 0.526] | **0.714**  **[0.699, 0.721]** | 3.13  (± 1.52) | n.s. |
| AD | MRMR | | 0.684  [0.678, 0.690] | 64.5  [63.9, 65.0] | 58.3  [57.6, 59.4] | 68.4  [67.5, 68.9] | 0.560  [0.543, 0.559] | 0.538  [0.530, 0.548] | 3.26  (± 1.55) | n.s. |
| RD | MRMR | | 0.579  [0.573, 0.584] | 61.3  [60.7, 61.8] | 41.7  [40.5, 42.4] | 73.7  [73.1, 74.3] | 0.455  [0.433, 0.449] | 0.500  [0.486, 0.506] | 2.97  (± 1.37) | n.s. |

**Supplementary Table S2** Test performance metrics of the classification models for each uni-modal feature set. This is shown for classification between subjects with major depressive disorder versus healthy controls (diagnosis) and positive versus negative outcome at 6-months follow-up ( outcome prediction). Abbreviations: sFC = static functional connectivity; nCC = number of coherence clusters; leadCoh = lead coherence; Act = activity; FA = fractional anisotropy; AD = axial diffusivity; RD = radial diffusivity; MRMR = minimum redundancy maximum relevance; CV-SVM = concave minimization and support vector machine; AUC = area under the curve; Acc = accuracy; Sens = sensitivity; Spec = specificity; Prec = precision; n.s. = non-significant (p-value > 0.05).

|  | **Uni-modal validation performance - Diagnosis** | | | | | | |
| --- | --- | --- | --- | --- | --- | --- | --- |
| **Feature set** | **Feature selection** | **AUC** | **Acc [%]** | **Sens [%]** | **Spec [%]** | **F1-score** | **Prec** |
| T_1_ | MRMR | 6.18•10^-1^ ± 7.84•10^-2^ | 5.20•10^-1^ ± 6.96•10^-2^ | 5.61•10^-1^ ± 4.74•10^-1^ | 4.78•10^-1^ ± 4.76•10^-1^ | 1.80•10^-1^ ± 3.16•10^-1^ | 5.28•10^-1^ ± 1.84•10^-1^ |
| T_2_ | CV-SVM | 4.92•10^-1^ ± 6.73•10^-2^ | 5.05•10^-1^ ± 5.51•10^-2^ | 5.60•10^-1^ ± 2.76•10^-1^ | 4.48•10^-1^ ± 2.72•10^-1^ | 4.23•10^-1^ ± 1.86•10^-1^ | 5.05•10^-1^ ± 7.93•10^-2^ |
| sFC | CV-SVM | 5.69•10^-1^ ± 6.80•10^-2^ | 5.41•10^-1^ ± 5.78•10^-2^ | 5.01•10^-1^ ± 2.50•10^-1^ | 5.83•10^-1^ ± 2.41•10^-1^ | 5.53•10^-1^ ± 1.77•10^-1^ | 5.74•10^-1^ ± 1.12•10^-1^ |
| nCC | MRMR | 6.20•10^-1^ ± 7.90•10^-2^ | 5.20•10^-1^ ± 5.86•10^-2^ | 6.17•10^-1^ ± 4.44•10^-1^ | 4.23•10^-1^ ± 4.53•10^-1^ | 2.71•10^-1^ ± 3.75•10^-1^ | 5.75•10^-1^ ± 2.20•10^-1^ |
| leadCoh | MRMR | 6.49•10^-1^ ± 1.02•10^-1^ | 5.40•10^-1^ ± 8.91•10^-2^ | 4.69•10^-1^ ± 4.46•10^-1^ | 6.13•10^-1^ ± 4.60•10^-1^ | 3.74•10^-1^ ± 4.31•10^-1^ | 6.49•10^-1^ ± 1.98•10^-1^ |
| Act | MRMR | 5.89•10^-1^ ± 5.60•10^-2^ | 4.99•10^-1^ ± 1.23•10^-2^ | 4.86•10^-1^ ± 5.00•10^-1^ | 5.13•10^-1^ ± 4.99•10^-1^ | 1.28•10^-2^ ± 7.48•10^-2^ | 4.63•10^-1^ ± 1.47•10^-1^ |
| sSW | MRMR | 6.97•10^-1^ ± 1.61•10^-1^ | 5.87•10^-1^ ± 1.48•10^-1^ | 5.24•10^-1^ ± 3.88•10^-1^ | 6.54•10^-1^ ± 3.58•10^-1^ | 5.35•10^-1^ ± 3.52•10^-1^ | 6.32•10^-1^ ± 2.56•10^-1^ |
| MD | CV-SVM | 5.59•10^-1^ ± 6.96•10^-2^ | 5.23•10^-1^ ± 5.41•10^-2^ | 5.21•10^-1^ ± 3.29•10^-1^ | 5.28•10^-1^ ± 3.25•10^-1^ | 4.56•10^-1^ ± 2.32•10^-1^ | 5.34•10^-1^ ± 9.31•10^-2^ |
| FA | CV-SVM | 5.53•10^-1^ ± 5.87•10^-2^ | 5.16•10^-1^ ± 4.17•10^-2^ | 4.20•10^-1^ ± 3.33•10^-1^ | 6.15•10^-1^ ± 3.32•10^-1^ | 4.91•10^-1^ ± 2.42•10^-1^ | 5.34•10^-1^ ± 1.28•10^-1^ |
| AD | MRMR | 6.82•10^-1^ ± 1.62•10^-1^ | 5.47•10^-1^ ± 1.17•10^-1^ | 4.62•10^-1^ ± 4.15•10^-1^ | 6.35•10^-1^ ± 4.16•10^-1^ | 4.34•10^-1^ ± 4.05•10^-1^ | 6.02•10^-1^ ± 2.74•10^-1^ |
| RD | CV-SVM | 5.95•10^-1^ ± 7.31•10^-2^ | 5.26•10^-1^ ± 4.90•10^-2^ | 4.89•10^-1^ ± 3.56•10^-1^ | 5.63•10^-1^ ± 3.50•10^-1^ | 4.40•10^-1^ ± 2.43•10^-1^ | 5.36•10^-1^ ± 1.10•10^-1^ |
|  | **Uni-modal validation performance – Outcome prediction** | | | | | | |
| **Feature set** | **Feature selection** | **AUC** | **Acc [%]** | **Sens [%]** | **Spec [%]** | **F1-score** | **Prec** |
| T_1_ | MRMR | 6.91•10^-1^ ± 1.13•10^-1^ | 5.58•10^-1^ ± 1.51•10^-1^ | 5.16•10^-1^ ± 4.50•10^-1^ | 5.89•10^-1^ ± 4.60•10^-1^ | 4.90•10^-1^ ± 1.97•10^-1^ | 6.05•10^-1^ ± 2.68•10^-1^ |
| T_2_ | CV-SVM | 6.03•10^-1^ ± 9.60•10^-2^ | 5.46•10^-1^ ± 1.08•10^-1^ | 4.50•10^-1^ ± 3.66•10^-1^ | 5.97•10^-1^ ± 3.60•10^-1^ | 4.22•10^-1^ ± 1.75•10^-1^ | 4.62•10^-1^ ± 1.88•10^-1^ |
| sFC | MRMR | 7.15•10^-1^ ± 1.30•10^-1^ | 5.94•10^-1^ ± 1.57•10^-1^ | 5.49•10^-1^ ± 4.47•10^-1^ | 6.23•10^-1^ ± 4.22•10^-1^ | 5.77•10^-1^ ± 1.81•10^-1^ | 5.48•10^-1^ ± 2.59•10^-1^ |
| nCC | CV-SVM | 6.42•10^-1^ ± 9.34•10^-2^ | 5.23•10^-1^ ± 1.15•10^-1^ | 5.01•10^-1^ ± 3.62•10^-1^ | 5.45•10^-1^ ± 3.83•10^-1^ | 4.25•10^-1^ ± 1.50•10^-1^ | 4.25•10^-1^ ± 1.87•10^-1^ |
| leadCoh | MRMR | 7.08•10^-1^ ± 1.76•10^-1^ | 5.94•10^-1^ ± 1.41•10^-1^ | 3.66•10^-1^ ± 4.30•10^-1^ | 7.40•10^-1^ ± 3.84•10^-1^ | 5.84•10^-1^ ± 1.70•10^-1^ | 4.83•10^-1^ ± 2.79•10^-1^ |
| Act | MRMR | 6.12•10^-1^ ± 8.07•10^-2^ | 5.48•10^-1^ ± 1.11•10^-1^ | 5.50•10^-1^ ± 3.44•10^-1^ | 5.51•10^-1^ ± 3.66•10^-1^ | 4.75•10^-1^ ± 1.34•10^-1^ | 4.88•10^-1^ ± 1.96•10^-1^ |
| sSW | CV-SVM | 6.94•10^-1^ ± 8.76•10^-2^ | 4.96•10^-1^ ± 1.12•10^-1^ | 5.33•10^-1^ ± 4.28•10^-1^ | 4.86•10^-1^ ± 4.38•10^-1^ | 4.49•10^-1^ ± 1.58•10^-1^ | 4.20•10^-1^ ± 2.23•10^-1^ |
| MD | CV-SVM | 6.24•10^-1^ ± 1.02•10^-1^ | 4.86•10^-1^ ± 1.10•10^-1^ | 5.69•10^-1^ ± 4.26•10^-1^ | 4.37•10^-1^ ± 4.25•10^-1^ | 5.12•10^-1^ ± 9.47•10^-2^ | 3.63•10^-1^ ± 2.03•10^-1^ |
| FA | MRMR | 6.72•10^-1^ ± 1.75•10^-1^ | 6.12•10^-1^ ± 1.27•10^-1^ | 3.31•10^-1^ ± 3.32•10^-1^ | 7.94•10^-1^ ± 3.00•10^-1^ | 4.66•10^-1^ ± 1.92•10^-1^ | 5.74•10^-1^ ± 3.08•10^-1^ |
| AD | MRMR | 7.01•10^-1^ ± 2.73•10^-1^ | 5.67•10^-1^ ± 1.83•10^-1^ | 4.65•10^-1^ ± 4.39•10^-1^ | 6.37•10^-1^ ± 4.15•10^-1^ | 5.74•10^-1^ ± 1.95•10^-1^ | 5.12•10^-1^ ± 3.44•10^-1^ |
| RD | MRMR | 7.65•10^-1^ ± 1.62•10^-1^ | 6.31•10^-1^ ± 1.68•10^-1^ | 5.57•10^-1^ ± 3.92•10^-1^ | 6.77•10^-1^ ± 3.62•10^-1^ | 5.99•10^-1^ ± 1.98•10^-1^ | 6.07•10^-1^ ± 2.48•10^-1^ |

**Supplementary Table S3** Validation performance metrics of the classification models for each uni-modal feature set. This is shown for classification between subjects with major depressive disorder versus healthy controls (diagnosis) and positive versus negative outcome at 6-months follow-up ( outcome prediction). Mean and standard deviation are shown as obtained over all inner loop iterations. Abbreviations: sFC = static functional connectivity; nCC = number of coherence clusters; leadCoh = lead coherence; Act = activity; FA = fractional anisotropy; AD = axial diffusivity; RD = radial diffusivity; MRMR = minimum redundancy maximum relevance; CV-SVM = concave minimization and support vector machine; AUC = area under the curve; Acc = accuracy; Sens = sensitivity; Spec = specificity; Prec = precision.

|  | **Multi-modal test performance - Diagnosis** | | | | | | | |
| --- | --- | --- | --- | --- | --- | --- | --- | --- |
| **Multi-modal method** | **Feature selection** | **AUC** | **Acc [%]** | **Sens [%]** | **Spec [%]** | **F1-score** | **Prec** | **k_optimal_** |
| Concatenation | MRMR | 0.559  [0.552, 0.560] | 55.6  [55.4, 56.2] | 62.5  [62.0, 63.1] | 48.4  [48.0, 49.1] | 0.588  [0.584, 0.593] | 0.556  [0.557, 0.567] | 3.38  (± 1.52) |
| Forced fusion | MRMR | 0.572  [0.565, 0.573] | 57.1  [56.5, 57.3] | 56.3  [55.3, 56.4] | 58.1  [57.4, 58.6] | 0.571  [0.559, 0.569] | 0.581  [0.573, 0.584] | 3.40  (± 1.30) |
| Ensemble (all features) | See uni-modal models | 0.621  [0.619, 0.626] | 61.9  [61.8, 62.5] | 53.1  [52.6, 53.7] | **71.0**  **[70.9, 71.9]** | 0.586  [0.578, 0.588] | 0.654  [0.650, 0.661]] | 2.96  (± 1.50) |
| Most optimal ensemble  (5 features) | See uni-modal models | **0.746**  **[0.741, 0.747]** | **74.6**  **[74.2, 74.8]** | **78.1**  **[77.6, 78.5]** | **71.0**  **[70.3, 71.3]** | **0.758**  **[0.749, 0.757]** | **0.735**  **[0.728, 0.737]** | 2.95  (± 1.50) |
|  | **Multi-modal test performance – Outcome prediction** | | | | | | | |
| **Multi-modal method** | **Feature selection** | **AUC** | **Acc [%]** | **Sens [%]** | **Spec [%]** | **F1-score** | **Prec** | **k_optimal_** |
| Concatenation | CV-SVM | 0.614  [0.612, 0.623] | 61.3  [61.0, 62.1] | 58.3  [57.8, 59.6] | 63.2  [62.8, 64.2] | 0.538  [0.523, 0.538] | 0.500  [0.492, 0.509] | 2.90  (± 1.33) |
| Forced fusion | CV-SVM | 0.460  [0.453, 0.464] | 41.9  [41.2, 42.3] | 33.3  [32.4, 34.1] | 47.4  [46.4, 47.7] | 0.308  [0.294, 0.308] | 0.286  [0.276, 0.291] | 2.71  (± 1.37) |
| Ensemble (all features) | See uni-modal models | 0.713  [0.704, 0.714] | 74.2  [73.2, 74.2] | 58.3  [56.8, 58.7] | 84.2  [83.5, 84.5] | 0.636  [0.614, 0.630] | 0.700  [0.692, 0.711] | 2.87  (± 1.45) |
| Most optimal ensemble  (top 5 features) | See uni-modal models | **0.932**  **[0.927, 0.934]** | **93.6**  **[93.1, 93.7]** | **91.7**  **[91.1, 92.1]** | **94.7**  **[94.2, 94.9]** | **0.917**  **[0.908, 0.916]** | **0.917**  **[0.910, 0.921]** | 2.96  (± 1.53) |

**Supplementary Table S4** Test performance metrics of the multi-modal classification models. Three methods were conducted to test the added value of multi-modal models. With concatenation, all feature sets were combined to one single feature vector and the same classification procedure was run as for all uni-modal approaches. With forced fusion, the top 10 feature elements of each feature set were selected based on group differences in the training folds, and added to a single feature vector. Subsequently, the same classification procedures as in the uni-modal approaches were resumed. For the ensemble models, the predicted labels from the uni-modal models were used. For each test subject, a majority voting algorithm determined the final label. The ensemble models were run for all (11) feature sets (3rd row) and for all possible combinations of 3, 5, 7 and 9 feature sets. The last row represents the most optimal ensemble model among these combinations. Abbreviations: AUC = area under the curve; Acc = accuracy; Sens = sensitivity; Spec = specificity; Prec = precision.

| **Improved multi-modal ensemble models - Diagnosis** | | | | | | |
| --- | --- | --- | --- | --- | --- | --- |
| **Feature sets** | **AUC** | **Acc [%]** | **Sens [%]** | **Spec [%]** | **F1** | **Prec** |
| T_1_, MD, FA | 0.730  [0.728, 0.735] | 73.0  [72.7, 73.4] | 71.9  [71.6, 72.6] | 74.2  [73.7, 74.6] | 0.730  [0.723, 0.731] | 0.742  [0.734, 0.744] |
| T_1_, sFC, nCC, Act, MD | 0.729  [0.728, 0.735] | 73.0  [72.8, 73.5] | 78.1  [77.9, 78.8] | 67.7  [67.3, 68.3] | 0.746  [0.741, 0.748] | 0.714  [0.709, 0.719] |
| T_1_, sFC, nCC, MD, FA | 0.745  [0.744, 0.751] | 74.6  [74.5, 75.2] | 78.1  [77.9, 78.8] | 71.0  [70.6, 71.6] | 0.758  [0.754, 0.761] | 0.735  [0.733, 0.743] |
| T_1_, sFC, nCC, MD, AD | 0.714  [0.709, 0.716] | 71.4  [70.9, 71.6] | 71.9  [71.2, 72.2] | 71.0  [70.3, 71.3] | 0.719  [0.710, 0.718] | 0.719  [0.713, 0.723] |
| T_1_, sFC, nCC, MD, RD | 0.730  [0.726, 0.733] | 73.0  [72.6, 73.3] | 75.0  [74.5, 75.4] | 71.0  [70.4, 71.5] | 0.738  [0.731, 0.738] | 0.727  [0.722, 0.731] |
| T_1_, sFC, Act, MD, FA | 0.714  [0.713, 0.720] | 71.4  [71.3, 72.0] | 75.0  [74.5, 75.4] | 67.7  [67.8, 68.8] | 0.727  [0.721, 0.729] | 0.706  [0.703, 0.713] |
| T_1_, sFC, nCC, leadCoh, MD, FA, RD | 0.716  [0.711, 0.718] | 71.4  [71.0, 71.7] | 62.5  [61.9, 63.0] | 80.6  [79.9, 80.8] | 0.690  [0.680, 0.688] | 0.769  [0.760, 0.771] |
| T_1_, sFC, nCC, Act, MD, FA, AD | 0.731  [0.726, 0.733] | 73.0  [72.6, 73.3] | 68.8  [68.2, 69.2] | 77.4  [76.8, 77.8] | 0.721  [0.713, 0.721] | 0.759  [0.753, 0.763] |
| T_1_, sFC, nCC, Act, MD, FA, RD | 0.715  [0.713, 0.720] | 71.4  [71.2, 71.9] | 68.8  [68.4, 69.4] | 74.2  [73.9, 74.9] | 0.710  [0.704, 0.712] | 0.733  [0.7300, 0.740] |
| T_1_, sFC, nCC, Act, MD, AD, RD | 0.715  [0.712, 0.719] | 71.4  [71.3, 71.8] | 68.8  [68.4, 69.4] | 74.2  [73.8, 74.7] | 0.710  [0.704, 0.712] | 0.733  [0.731, 0.740] |
| T_1_, sFC, nCC, sSW, MD, FA, AD | 0.715  [0.709, 0.716] | 71.4  [70.9, 71.6] | 68.8  [68.5, 69.5] | 74.2  [73.1, 74.0] | 0.710  [0.700, 0.708] | 0.733  [0.721, 0.731] |
| **Improved multi-modal ensemble models – Outcome prediction** | | | | | | |
| **Feature sets** | **AUC** | **Acc [%]** | **Sens [%]** | **Spec [%]** | **F1** | **Prec** |
| sSW, MD, FA | 0.864  [0.860, 0.869] | 87.1  [86.7, 87.5] | 83.3  [82.7, 84.1] | 89.5  [89.0, 89.9] | 0.833  [0.823, 0.834] | 0.833  [0.829, 0.843] |
| T_1_, nCC, leadCoh, Act, sSW | 0.921  [0.919, 0.925] | 90.3  [90.1, 90.7] | 100  [100,100] | 84.2  [83.9, 84.9] | 0.889  [0.882, 0.890] | 0.800  [0.795, 0.809] |
| T_1_, nCC, leadCoh, sSW, FA | 0.890  [0.885, 0.893] | 90.3  [89.9, 90.6] | 83.3  [82.4, 83.8] | 94.7  [94.4, 95.0] | 0.870  [0.857, 0.868] | 0.909  [0.902, 0.913] |
| T_1_, nCC, leadCoh, sSW, AD | 0.879  [0.877, 0.884] | 87.1  [86.9, 87.6] | 91.7  [91.2, 92.1] | 84.2  [84.0, 85.0] | 0.846  [0.837, 0.847] | 0.786  [0.781, 0.794] |
| T_1_, leadCoh, Act, sSW, FA | 0.906  [0.905, 0.911] | 90.3  [90.3, 90.9] | 91.7  [91.7, 92.7] | 89.5  [89.1, 90.0] | 0.880  [0.873, 0.882] | 0.846  [0.839, 0.852] |
| T_1_, leadCoh, sSW, MD, FA | 0.864  [0.864, 0.872] | 87.1  [87.0, 87.8] | 83.3  [83.3, 84.6] | 89.5  [89.1, 90.0] | 0.833  [0.827, 0.837] | 0.833  [0.830, 0.843] |
| T_1_, leadCoh, sSW, FA, AD | 0.864  [0.858, 0.867] | 87.1  [86.5, 87.2] | 83.3  [82.5, 83.9] | 89.5  [88.8, 89.8] | 0.833  [0.819, 0.831] | 0.833  [0.825, 0.839] |
| nCC, leadCoh, Act, sSW, MD | 0.879  [0.874, 0.882] | 87.1  [86.4, 87.2] | 91.7  [91.5, 92.5] | 84.2  [83.1, 84.2] | 0.846  [0.832, 0.843] | 0.786  [0.771, 0.786] |
| nCC, leadCoh, Act, sSW, RD | 0.864  [0.859, 0.867] | 87.1  [86.7, 87.4] | 83.3  [82.5, 83.9] | 89.5  [89.0, 89.9] | 0.833  [0.823, 0.834] | 0.833  [0.830, 0.844] |
| leadCoh, sSW, MD, FA, AD | 0.932  [0.927, 0.934] | 93.5  [93.1, 93.7] | 91.7  [91.1, 92.1] | 94.7  [94.2, 94.9] | 0.917  [0.906, 0.914] | 0.917  [0.906, 0.917] |
| T_1_, T_2_, nCC, leadCoh, Act, sSW, FA | 0.875  [0.871, 0.878] | 90.3  [89.9, 90.5] | 75.0  [74..1, 757] | 100  [100, 100] | 0.857  [0.845, 0.856] | 1.00  [100, 100] |
| T_1_, sFC, nCC, leadCoh, sSW, FA, AD | 0.864  [0.863, 0.871] | 87.1  [87.0, 87.7] | 83.3  [83.2, 84.6] | 89.5  [89.1, 90.0] | 0.833  [0.826, 0.837] | 0.833  [0.830, 0.844] |
| T_1_, nCC, leadCoh, Act, sSW, MD, FA | 0.906  [0.901, 0.908] | 90.3  [89.9, 90.6] | 91.7  [90.9, 91.9] | 89.5  [89.1, 89.9] | 0.880  [0.869, 0.878] | 0.846  [0.838, 0.851] |
| T_1_, nCC, leadCoh, Act, sSW, FA, AD | 0.917  [0.911, 0.917] | 93.5  [93.1, 93.6] | 83.3  [82.1, 83.5] | 100  [100, 100] | 0.909  [0.898, 0.906] | 1.00  [100, 100] |
| T_1_, nCC, leadCoh, Act, sSW, FA, RD | 0.875  [0.867, 0.875] | 90.3  [89.6, 90.3] | 75.0  [73.4, 75.0] | 100  [100, 100] | 0.857  [0.840, 0.851] | 1.00  [100, 100] |
| T_1_, nCC, leadCoh, sSW, MD, FA, AD | 0.890  [0.887, 0.895] | 90.3  [90.0, 90.6] | 83.3  [82.8, 84.2] | 94.7  [94.3, 95.0] | 0.870  [0.860, 0.870] | 0.909  [0.903, 0.914] |
| T_1_, nCC, leadCoh, sSW, FA, AD, RD | 0.875  [0.871, 0.879] | 90.3  [90.0, 90.6] | 75.0  [74.3, 75.9] | 100  [100, 100] | 0.857  [0.846, 0.857] | 1.00  [100, 100] |

**Supplementary Table S5** All multi-modal ensemble models that improved the AUC of the most optimal uni-modal model for diagnosis and outcome prediction. T1 and MD feature sets were represented in 100% of the diagnostic models whereas sSW was represented in all outcome models. Abbreviations: AUC = area under the curve; Acc = accuracy; Sens = sensitivity; Spec = specificity; Prec = precision; sFC = static functional connectivity; nCC = number of coherence clusters; leadCoh = lead coherence; Act = activity; FA = fractional anisotropy; AD = axial diffusivity; RD = radial diffusivity.

| **Outer fold feature element contribution – Diagnosis** | | | | | | |
| --- | --- | --- | --- | --- | --- | --- |
| **Feature element** | **p-val** | **Mean ± std MDD** | | **Mean ± std HC** | **% outer fold** | **Statistical test** |
| CT_R.IPG | 2.23•10^-1^ | | 2.40•10^0^ ± 9.93•10^-2^ | 2.44•10^0^ ± 1.00•10^-1^ | 7.78•10^1^ | t-test |
| CT_R.POP | 5.79•10^-1^ | | 2.52•10^0^ ± 1.53•10^-1^ | 2.55•10^0^ ± 1.46•10^-1^ | 6.98•10^1^ | t-test |
| CT_R.ICG | 5.97•10^-1^ | | 2.31•10^0^ ± 1.60•10^-1^ | 2.35•10^0^ ± 1.76•10^-1^ | 4.76•10^1^ | MW-U test |
| Vol_L.Sb | 1.21•10^-2^ | | 2.10•10^2^ ± 2.77•10^1^ | 2.27•10^2^ ± 2.94•10^1^ | 6.03•10^1^ | MW-U test |
| Vol_R.PSb | 1.76•10^-1^ | | 1.38•10^2^ ± 2.41•10^1^ | 1.46•10^2^ ± 2.14•10^1^ | 4.76•10^1^ | t-test |
| Vol_L.PSb | 3.11•10^-2^ | | 1.50•10^2^ ± 2.42•10^1^ | 1.64•10^2^ ± 2.65•10^1^ | 4.13•10^1^ | t-test |
| sFC_CN_aDMN | 4.50•10^-2^ | | 3.95•10^-2^ ± 1.98•10^-1^ | -6.65•10^-2^ ± 2.13•10^-1^ | 4.76•10^1^ | t-test |
| sFC_DAN_lFPN | 1.77•10^-2^ | | -8.36•10^-2^ ± 2.39•10^-1^ | -2.20•10^-1^ ± 2.26•10^-1^ | 3.49•10^1^ | MW-U test |
| sFC_CN_lFPN | 3.00•10^-2^ | | -2.53•10^-2^ ± 1.89•10^-1^ | 7.59•10^-2^ ± 1.72•10^-1^ | 3.33•10^1^ | t-test |
| nCC_SMN_latSMN | 5.40•10^-3^ | | 1.43•10^1^ ± 3.72•10^0^ | 1.18•10^1^ ± 4.16•10^0^ | 9.84•10^1^ | MW-U test |
| nCC_lFPN_latVN | 7.27•10^-3^ | | 1.48•10^1^ ± 2.19•10^0^ | 1.32•10^1^ ± 2.52•10^0^ | 8.10•10^1^ | t-test |
| nCC_rFPN_SN | 3.36•10^-2^ | | 1.43•10^1^ ± 2.50•10^0^ | 1.29•10^1^ ± 2.51•10^0^ | 6.35•10^1^ | MW-U test |
| leadCoh_DMN_SMN | 1.08•10^-2^ | | 7.66•10^0^ ± 6.75•10^0^ | 3.82•10^0^ ± 3.11•10^0^ | 7.78•10^1^ | MW-U test |
| leadCoh_lFPN_AN | 2.22•10^-3^ | | 1.15•10^1^ ± 8.24•10^0^ | 6.79•10^0^ ± 6.84•10^0^ | 7.30•10^1^ | MW-U test |
| leadCoh_SMN_latVN | 3.79•10^-2^ | | 1.00•10^1^ ± 9.85•10^0^ | 6.54•10^0^ ± 7.27•10^0^ | 6.67•10^1^ | MW-U test |
| Act_Fusiform_R_  Faces_Shapes | 4.82•10^-3^ | | 1.50•10^0^ ± 8.19•10^-1^ | 2.44•10^0^ ± 1.61•10^0^ | 1.00•10^2^ | t-test |
| Act_ACC_Faces_Rest | 3.73•10^-2^ | | -6.42•10^-1^ ± 1.06•10^0^ | -1.09•10^0^ ± 1.19•10^0^ | 3.49•10^1^ | MW-U test |
| Act_ParaHippocampal_L_  Faces_Rest | 1.13•10^-1^ | | -1.65•10^-1^ ± 7.54•10^-1^ | -4.80•10^-1^ ± 8.00•10^-1^ | 3.33•10^1^ | t-test |
| sSW_R.PaCG - R.PA | 3.19•10^-3^ | | 8.02•10^-1^ ± 4.50•10^-1^ | 5.57•10^-1^ ± 5.90•10^-1^ | 7.78•10^1^ | MW-U test |
| sSW_R.POR - R.LOFG | 1.46•10^-3^ | | 2.28•10^0^ ± 1.53•10^0^ | 3.52•10^0^ ± 1.62•10^0^ | 4.13•10^1^ | MW-U test |
| sSW_L.PCAL - L.CU | 2.34•10^-4^ | | 3.00•10^1^ ± 8.85•10^0^ | 2.21•10^1^ ± 6.92•10^0^ | 3.81•10^1^ | t-test |
| MD_R.SMG - R.PCG | 3.97•10^-3^ | | 5.11•10^-4^ ± 1.38•10^-4^ | 5.68•10^-4^ ± 3.95•10^-5^ | 6.67•10^1^ | MW-U test |
| MD_R.AC - L.SMG | 3.26•10^-3^ | | 4.20•10^-4^ ± 3.92•10^-4^ | 1.55•10^-4^ ± 2.67•10^-4^ | 4.60•10^1^ | MW-U test |
| MD_R.PrCG - L.RACG | 1.66•10^-3^ | | 3.01•10^-4^ ± 2.89•10^-4^ | 5.50•10^-4^ ± 1.61•10^-4^ | 4.29•10^1^ | MW-U test |
| FA_R.AC - R.PA | 1.60•10^-3^ | | 2.54•10^-1^ ± 8.43•10^-2^ | 3.15•10^-1^ ± 6.04•10^-2^ | 3.97•10^1^ | MW-U test |
| FA_R.FP - L.RMFG | 1.06•10^-3^ | | 3.53•10^-1^ ± 2.26•10^-1^ | 4.82•10^-1^ ± 1.34•10^-1^ | 3.33•10^1^ | MW-U test |
| FA_R.RACG - L.RMFG | 1.04•10^-3^ | | 3.43•10^-1^ ± 2.21•10^-1^ | 4.85•10^-1^ ± 9.92•10^-2^ | 3.02•10^1^ | MW-U test |
| AD_R.PrCG - L.RACG | 2.82•10^-4^ | | 4.87•10^-4^ ± 4.67•10^-4^ | 9.07•10^-4^ ± 2.69•10^-4^ | 7.78•10^1^ | MW-U test |
| AD_L.AM - L.TTG | 1.13•10^-3^ | | 6.77•10^-4^ ± 4.05•10^-4^ | 9.03•10^-4^ ± 1.75•10^-4^ | 4.44•10^1^ | MW-U test |
| AD_L.PCG - L.POP | 2.66•10^-3^ | | 8.04•10^-4^ ± 1.53•10^-4^ | 8.59•10^-4^ ± 3.65•10^-5^ | 4.13•10^1^ | MW-U test |
| RD_L.TTG - L.MTG | 4.53•10^-3^ | | 3.93•10^-4^ ± 7.61•10^-5^ | 4.28•10^-4^ ± 3.36•10^-5^ | 5.40•10^1^ | MW-U test |
| RD_R.STG - R.MOFG | 2.91•10^-3^ | | 3.88•10^-4^ ± 1.62•10^-4^ | 4.93•10^-4^ ± 1.36•10^-4^ | 5.08•10^1^ | MW-U test |
| RD_R.RACG - R.AM | 4.47•10^-3^ | | 1.67•10^-4^ ± 2.11•10^-4^ | 3.08•10^-4^ ± 1.90•10^-4^ | 3.65•10^1^ | MW-U test |

**Supplementary Table S6** Feature element model contributions for diagnosis as expressed in % of occurrence in the outer loop. Abbreviations: std = standard deviation; MDD = major depressive disorder; HC = healthy controls; MW-U = Mann-Whitney U; CT = cortical thickness; Vol = volume; sFC = static functional connectivity; nCC = number of coherence clusters; leadCoh = lead coherence; Act = activity; sSW = sum of streamline weights; MD = mean diffusivity; FA = fractional anisotropy; AD = axial diffusivity; RD = radial diffusivity; IPG = inf par gyrus; POP = pars opercularis; ICG = isthmus cingulate gyrus; Sb = subiculum body; PSb = presubiculum body; CN = cerebellum network; aDMN = ant default mode network; DAN = dorsal attention network; lFPN = left frontoparietal network; SMN = sensorimotor network; latSMN = lat sensorimotor network; latVN = lat visual network; rFPN = right frontoparietal network; SN = salience network; DMN = default mode network; AN = auditory network; Fusiform_R_Faces_Shapes = r fusiform faces-shapes; ACC_Faces_Rest = ant CC faces-rest; ParaHippocampal_L_Faces_Rest = l parahippocampus faces-rest; PaCG = paracentral gyrus; PA = pallidum; POR = pars orbitalis; LOFG = lat orbitofrontal gyrus; PCAL = pericalcarine; CU = cuneus; SMG = supramarginal gyrus; PCG = post cingulate gyrus; AC = accumbens area; PrCG = precentral gyrus; RACG = rost ant cingulate gyrus; FP = front pole; RMFG = rost mid front gyrus; AM = amygdala; TTG = transverse temp gyrus; MTG = mid temp gyrus; STG = sup temp gyrus; MOFG = med orbitofrontal gyrus.

| **Outer fold feature element contribution – Outcome prediction** | | | | | |
| --- | --- | --- | --- | --- | --- |
| **Feature element** | **p-val** | **Mean ± std NCO** | **Mean ± std PCO** | **% outer fold** | **Statistical test** |
| CT_R.MTG | 3.46•10^-1^ | 2.77•10^0^ ± 1.27•10^-1^ | 2.81•10^0^ ± 1.40•10^-1^ | 9.69•10^1^ | t-test |
| CT_L.CU | 8.47•10^-2^ | 1.85•10^0^ ± 1.01•10^-1^ | 1.92•10^0^ ± 1.16•10^-1^ | 6.88•10^1^ | MW-U test |
| CT_R.ICG | 2.86•10^-1^ | 2.29•10^0^ ± 1.56•10^-1^ | 2.36•10^0^ ± 1.71•10^-1^ | 4.06•10^1^ | t-test |
| Vol_R.PS | 2.90•10^-1^ | 6.48•10^1^ ± 1.02•10^1^ | 6.00•10^1^ ± 1.46•10^1^ | 6.25•10^1^ | t-test |
| Vol_L.MLHPb | 5.63•10^-1^ | 2.15•10^2^ ± 3.82•10^1^ | 2.07•10^2^ ± 3.22•10^1^ | 3.75•10^1^ | t-test |
| Vol_L.CA4h | 7.00•10^-1^ | 1.24•10^2^ ± 1.96•10^1^ | 1.19•10^2^ ± 1.85•10^1^ | 3.12•10^1^ | MW-U test |
| sFC_CN_SMN | 1.21•10^-1^ | -2.26•10^-1^ ± 2.70•10^-1^ | -6.36•10^-2^ ± 2.85•10^-1^ | 9.38•10^1^ | t-test |
| sFC_latVN_lFPN | 9.26•10^-2^ | -2.19•10^-1^ ± 1.87•10^-1^ | -1.01•10^-1^ ± 1.76•10^-1^ | 8.12•10^1^ | t-test |
| sFC_oVN_lFPN | 9.55•10^-1^ | -6.58•10^-2^ ± 1.73•10^-1^ | -6.23•10^-2^ ± 1.66•10^-1^ | 5.31•10^1^ | t-test |
| nCC_DAN_SMN | 7.49•10^-2^ | 1.31•10^1^ ± 2.82•10^0^ | 1.50•10^1^ ± 2.92•10^0^ | 3.44•10^1^ | t-test |
| nCC_DMN_CN | 7.99•10^-1^ | 1.39•10^1^ ± 3.43•10^0^ | 1.36•10^1^ ± 3.06•10^0^ | 3.12•10^1^ | t-test |
| nCC_SMN_latVN | 7.57•10^-1^ | 1.46•10^1^ ± 2.69•10^0^ | 1.49•10^1^ ± 3.29•10^0^ | 3.12•10^1^ | t-test |
| leadCoh_lFPN_pVN | 6.50•10^-2^ | 1.22•10^1^ ± 9.12•10^0^ | 7.54•10^0^ ± 8.82•10^0^ | 7.81•10^1^ | MW-U test |
| leadCoh_DAN_AN | 2.65•10^-1^ | 6.42•10^0^ ± 6.72•10^0^ | 9.17•10^0^ ± 6.60•10^0^ | 7.19•10^1^ | MW-U test |
| leadCoh_oVN_pVN | 7.61•10^-1^ | 1.17•10^1^ ± 8.50•10^0^ | 9.35•10^0^ ± 4.93•10^0^ | 4.38•10^1^ | MW-U test |
| lAmyg_Faces_Shapes | 1.83•10^-1^ | 1.40•10^0^ ± 1.02•10^0^ | 9.40•10^-1^ ± 7.35•10^-1^ | 4.06•10^1^ | t-test |
| rAmyg_Faces_Rest | 4.02•10^-1^ | 1.26•10^0^ ± 9.74•10^-1^ | 9.81•10^-1^ ± 7.11•10^-1^ | 3.75•10^1^ | t-test |
| rAmyg_Faces_Shapes | 5.86•10^-2^ | 1.47•10^0^ ± 1.04•10^0^ | 8.48•10^-1^ ± 4.20•10^-1^ | 3.75•10^1^ | t-test |
| sSW_R.IN - R.PHIG | 4.53•10^-1^ | 4.30•10^-2^ ± 5.94•10^-2^ | 1.94•10^-2^ ± 1.52•10^-2^ | 6.25•10^1^ | MW-U test |
| sSW_R.POR - L.BSTS | 3.43•10^-1^ | 1.65•10^-4^ ± 2.72•10^-4^ | 7.94•10^-5^ ± 1.77•10^-4^ | 6.25•10^1^ | MW-U test |
| sSW_R.PHIG - L.IN | 6.58•10^-1^ | 5.85•10^-4^ ± 8.45•10^-4^ | 8.24•10^-4^ ± 1.47•10^-3^ | 1.56•10^1^ | MW-U test |
| MD_R.SPG - R.RACG | 5.03•10^-1^ | 5.77•10^-4^ ± 2.63•10^-5^ | 5.57•10^-4^ ± 2.19•10^-4^ | 6.25•10^1^ | MW-U test |
| MD_R.PCAL - R.PHIG | 5.56•10^-1^ | 5.94•10^-4^ ± 2.20•10^-4^ | 6.12•10^-4^ ± 2.32•10^-4^ | 5.00•10^1^ | MW-U test |
| MD_R.POR - L.BSTS | 2.76•10^-1^ | 2.41•10^-4^ ± 2.91•10^-4^ | 1.40•10^-4^ ± 2.53•10^-4^ | 2.81•10^1^ | MW-U test |
| FA_L.STG - L.PaCG | 6.41•10^-1^ | 4.80•10^-1^ ± 2.63•10^-2^ | 4.48•10^-1^ ± 1.41•10^-1^ | 6.56•10^1^ | MW-U test |
| FA_R.PCG - R.AC | 7.92•10^-1^ | 4.33•10^-1^ ± 1.23•10^-1^ | 3.90•10^-1^ ± 1.85•10^-1^ | 5.31•10^1^ | MW-U test |
| FA_R.POR - L.BSTS | 3.68•10^-1^ | 2.26•10^-1^ ± 2.74•10^-1^ | 1.35•10^-1^ ± 2.45•10^-1^ | 2.81•10^1^ | MW-U test |
| AD_L.PHIG - L.LOFG | 5.82•10^-1^ | 7.90•10^-4^ ± 4.91•10^-4^ | 8.41•10^-4^ ± 4.09•10^-4^ | 7.81•10^1^ | MW-U test |
| AD_L.LOFG - L.CMFG | 8.08•10^-1^ | 6.89•10^-4^ ± 3.15•10^-4^ | 6.56•10^-4^ ± 3.08•10^-4^ | 5.62•10^1^ | MW-U test |
| AD_R.POR - L.BSTS | 2.55•10^-1^ | 4.11•10^-4^ ± 4.98•10^-4^ | 2.39•10^-4^ ± 4.33•10^-4^ | 2.81•10^1^ | MW-U test |
| RD_R.PCU - L.STG | 3.20•10^-1^ | 3.68•10^-4^ ± 2.56•10^-5^ | 3.35•10^-4^ ± 1.06•10^-4^ | 5.94•10^1^ | MW-U test |
| RD_R.SPG - R.RACG | 9.84•10^-1^ | 3.97•10^-4^ ± 2.77•10^-5^ | 3.93•10^-4^ ± 1.56•10^-4^ | 5.31•10^1^ | MW-U test |
| RD_R.TH - L.LOFG | 6.12•10^-1^ | 4.10•10^-4^ ± 1.15•10^-4^ | 3.83•10^-4^ ± 1.25•10^-4^ | 5.31•10^1^ | MW-U test |

**Supplementary Table S7** Feature element model contributions for outcome prediction as expressed in % of occurrence in the outer loop. Abbreviations: std = standard deviation; NCO/PCO = negative/positive outcome; MW-U = Mann-Whitney U; CT = cortical thickness; Vol = volume; sFC = static functional connectivity; nCC = number of coherence clusters; leadCoh = lead coherence; Act = activity; sSW = sum of streamline weights; MD = mean diffusivity; FA = fractional anisotropy; AD = axial diffusivity; RD = radial diffusivity; MTG = mid temp gyrus; CU = cuneus; ICG = isthmus cingulate gyrus; PS = parasubiculum; MLHPb = molecular layer hp body; CA4h = ca4 head; CN = cerebellum network; SMN = sensorimotor network; latVN = lat visual network; lFPN = left frontoparietal network; oVN = occ visual network; DAN = dorsal attention network; DMN = default mode network; pVN = prim visual network; AN = auditory network; lAmyg_Faces_Shapes = l amygdala faces-shapes; rAmyg_Faces_Rest = r hippocampus faces-rest; rAmyg_Faces_Shapes = r hippocampus faces-shapes; IN = insula; PHIG = parahippocampal gyrus; POR = pars orbitalis; BSTS = banks of sup temp sulcus gyrus; SPG = sup par gyrus; RACG = rost ant cingulate gyrus; PCAL = pericalcarine; STG = sup temp gyrus; PaCG = paracentral gyrus; PCG = post cingulate gyrus; AC = accumbens area; LOFG = lat orbitofrontal gyrus; CMFG = caud mid frontal gyrus; PCU = precuneus; TH = thalamus.

| **Feature element importance (β-coefficient) - Diagnosis** | | |
| --- | --- | --- |
| **Feature element** | **Sum of β-coefficient** | **Normalized β-coefficient** |
| CT_R.POP | 3.85•10^1^ | 1.00•10^0^ |
| CT_R.IPG | 2.04•10^1^ | 6.13•10^-1^ |
| CT_R.CMFG | 6.72•10^1^ | 2.08•10^-1^ |
| Vol_L.Sb | 2.84•10^1^ | 1.00•10^0^ |
| Vol_R.CA3b | 1.81•10^1^ | 6.38•10^-1^ |
| Vol_L.PSb | 1.35•10^1^ | 5.07•10^-1^ |
| sFC_CN_aDMN | 2.18•10^1^ | 1.00•10^0^ |
| sFC_CN_lFPN | 1.64•10^1^ | 7.64•10^-1^ |
| sFC_DAN_lFPN | 1.45•10^1^ | 7.85•10^-1^ |
| nCC_SMN_latSMN | 4.06•10^1^ | 1.00•10^0^ |
| nCC_lFPN_latVN | 3.58•10^1^ | 9.45•10^-1^ |
| nCC_rFPN_SN | 1.97•10^1^ | 5.40•10^-1^ |
| leadCoh_DMN_SMN | 4.31•10^1^ | 1.00•10^0^ |
| leadCoh_lFPN_AN | 4.22•10^1^ | 9.59•10^-1^ |
| leadCoh_SMN_latVN | 1.71•10^1^ | 4.06•10^-1^ |
| Act_Fusiform_R_  Faces_Shapes | 6.11•10^1^ | 1.00•10^0^ |
| Act_vmPFC_Faces_Rest | 1.17•10^1^ | 1.96•10^-1^ |
| Act_ParaHippocampal_L_  Faces_Rest | 9.05•10^0^ | 1.55•10^-1^ |
| sSW_R.PaCG - R.PA | 2.17•10^1^ | 1.00•10^0^ |
| sSW_L.PCAL - L.CU | 2.09•10^1^ | 8.74•10^-1^ |
| sSW_R.POR - R.LOFG | 2.01•10^1^ | 8.85•10^-1^ |
| MD_R.SMG - R.PCG | 4.91•10^1^ | 1.00•10^0^ |
| MD_R.AC - L.SMG | 2.78•10^1^ | 6.86•10^-1^ |
| MD_R.PrCG - L.RACG | 2.58•10^1^ | 6.74•10^-1^ |
| FA_R.AC - R.PA | 1.88•10^1^ | 1.00•10^0^ |
| FA_R.PrCG - L.RACG | 1.51•10^1^ | 8.84•10^-1^ |
| FA_R.RACG - L.RMFG | 1.36•10^1^ | 7.87•10^-1^ |
| AD_R.PrCG - L.RACG | 4.86•10^1^ | 1.00•10^0^ |
| AD_L.PCG - L.POP | 3.09•10^1^ | 4.66•10^-1^ |
| AD_L.AM - L.TTG | 1.67•10^1^ | 3.42•10^-1^ |
| RD_L.TTG - L.MTG | 4.22•10^1^ | 1.00•10^0^ |
| RD_R.STG - R.MOFG | 2.82•10^1^ | 7.24•10^-1^ |
| RD_R.AC – L.SMG | 1.98•10^1^ | 5.43•10^-1^ |

**Supplementary Table S8** Top 3 elements for each feature ranked by β-coefficients forming the hyperplane of the linear support vector machine models in the diagnosis classification. The higher a β-coefficient, the more effect on the hyperplane and thus classification outcome. Note that feature element values were normalized before classification in order to eliminate differences in absolute value between feature elements. The middle column is the sum of β-coefficients for that feature element over all outer loop folds. The right column shows the normalized sum of β-coefficients. Normalization took place in each separate model of the outer loop folds, followed by summation. This was done in order to control for the effect of potential β-coefficient outliers. Abbreviations: CT = cortical thickness; Vol = volume; sFC = static functional connectivity; nCC = number of coherence clusters; leadCoh = lead coherence; Act = activity; sSW = sum of streamline weights; MD = mean diffusivity; FA = fractional anisotropy; AD = axial diffusivity; R. = right; L. = left; a = anterior; lat = lateral; rost = rostral; mid = middle; front = frontal; temp = temporal; sup = superior; RD = radial diffusivity; POP = pars opercularis; IPG = inf par gyrus; CMFG = caudal mid frontal gyrus; Sb = subiculum body; CA3b = CA3 body; PSb = presubiculum body; CN = cerebellum network; DMN = default mode network; lFPN = left frontoparietal network; DAN = dorsal attention network; SMN = sensorimotor network; SMN = sensorimotor network; VN = visual network; rFPN = right frontoparietal network; SN = salience network; AN = auditory network; vmPFC = ventromedial prefrontal cortex; PaCG = paracentral gyrus; PA = pallidum; PCAL = pericalcarine; CU = cuneus; POR = pars orbitalis; LOFG = lat orbitofrontal gyrus; SMG = supramarginal gyrus; PCG = post cingulate gyrus; AC = accumbens area; PrCG = precentral gyrus; RACG = rost a cingulate gyrus; RMFG = rost mid front gyrus; AM = amygdala; TTG = transverse temp gyrus; MTG = mid temp gyrus; STG = sup temp gyrus; MOFG = med orbitofrontal gyrus.

| **Feature element importance (β-coefficient) – Outcome prediction** | | |
| --- | --- | --- |
| **Feature element** | **Cumulative of β-coefficients** | **Cumulative of β-coefficients (normalized)** |
| CT_R.MTG | 2.34•10^1^ | 1.00•10^0^ |
| CT_L.CU | 1.28•10^1^ | 5.71•10^-1^ |
| CT_R.ICG | 9.57•10^0^ | 4.10•10^-1^ |
| Vol_R.PS | 2.33•10^1^ | 1.00•10^0^ |
| Vol_L.MN | 9.38•10^0^ | 4.85•10^-1^ |
| Vol_L.CA4h | 7.68•10^0^ | 4.17•10^-1^ |
| sFC_CN_SMN | 2.39•10^1^ | 1.00•10^0^ |
| sFC_latSMN_aDMN | 1.84•10^1^ | 8.30•10^-1^ |
| sFC_latVN_lFPN | 1.01•10^1^ | 4.73•10^-1^ |
| nCC_AN_SN | 1.28•10^1^ | 1.00•10^0^ |
| nCC_rFPN_latVN | 1.16•10^1^ | 8.32•10^-1^ |
| nCC_DMN_CN | 5.95•10^0^ | 4.59•10^-1^ |
| leadCoh_lFPN_pVN | 3.06•10^1^ | 1.00•10^0^ |
| leadCoh_DAN_AN | 1.73•10^1^ | 6.39•10^-1^ |
| leadCoh_AN_CN | 7.45•10^0^ | 2.93•10^-1^ |
| lHip_Faces_Shapes | 2.33•10^1^ | 1.00•10^0^ |
| lAmyg_Faces_Shapes | 9.43•10^0^ | 4.23•10^-1^ |
| rHip_Faces_shapes | 3.08•10^0^ | 1.40•10^-1^ |
| sSW_R.IN - L.STG | 2.63•10^1^ | 1.00•10^0^ |
| sSW_R.POR - L.BSTS | 1.42•10^1^ | 6.75•10^-1^ |
| sSW_R.PrCG – R.AC | 1.12•10^1^ | 6.10•10^-1^ |
| MD_R.SPG - R.RACG | 2.40•10^1^ | 1.00•10^0^ |
| MD_R.PCAL - R.PHIG | 1.36•10^1^ | 6.08•10^-1^ |
| MD_R.POR - L.BSTS | 1.12•10^1^ | 5.86•10^-1^ |
| FA_R.POR - L.BSTS | 2.45•10^1^ | 1.00•10^0^ |
| FA_L.SMG – L.STG | 1.64•10^1^ | 6.03•10^-1^ |
| FA_L.STG - L.PaCG | 1.55•10^1^ | 6.15•10^-1^ |
| AD_L.PHIG - L.LOFG | 3.07•10^1^ | 1.00•10^0^ |
| AD_L.LOFG - L.CMFG | 1.14•10^1^ | 3.72•10^-1^ |
| AD_R.POR - L.BSTS | 9.17•10^0^ | 4.11•10^-1^ |
| RD_R.SPG - R.RACG | 3.25•10^1^ | 1.00•10^0^ |
| RD_R.PCU - L.STG | 1.21•10^1^ | 4.55•10^-1^ |
| RD_R.PCU - R.MOFG | 8.84•10^0^ | 3.20•10^-1^ |

**Supplementary Table S9** Top 3 elements for each feature ranked by β-coefficients forming the hyperplane of the linear support vector machine models in the outcome prediction. The higher a β-coefficient, the more effect on the hyperplane and thus classification outcome. Note that feature element values were normalized before classification in order to eliminate differences in absolute value between feature elements. The middle column is the sum of β-coefficients for that feature element over all outer loop folds. The right column shows the normalized sum of β-coefficients. Normalization took place in each separate model of the outer loop folds, followed by summation. This was done in order to control for the effect of potential β-coefficient outliers. Abbreviations: CT = cortical thickness; Vol = volume; sFC = static functional connectivity; nCC = number of coherence clusters; leadCoh = lead coherence; Act = activity; sSW = sum of streamline weights; MD = mean diffusivity; FA = fractional anisotropy; AD = axial diffusivity; R. = right; L. = left; a = anterior; lat = lateral; rost = rostral; mid = middle; front = frontal; temp = temporal; sup = superior; RD = radial diffusivity; MTG = mid temp gyrus; CU = cuneus; ICG = isthmus cingulate gyrus; PS = parasubiculum; MN = medial nucleus; CA4h = ca4 head; CN = cerebellum network; SMN = sensorimotor network; DMN = default mode network; VN = visual network; l/rFPN = left/right frontoparietal network; AN = auditory network; SN = salience network; pVN = primal visual network; DAN = dorsal attention network; l/rHip = left/right hippocampus; lAmyg = left amygdala; IN = insula; STG = sup temp gyrus; POR = pars orbitalis; BSTS = banks of sup temp sulcus gyrus; PrCG = precentral gyrus; AC = accumbens area; SPG = sup par gyrus; RACG = rost ant cingulate gyrus; PCAL = pericalcarine; PHIG = parahippocampal gyrus; SMG = supramarginal gyrus; PaCG = paracentral gyrus; LOFG = lat orbitofrontal gyrus; CMFG = caud mid frontal gyrus; PCU = precuneus; MOFG = med orbitofrontal gyrus.

| **Highest significant differences – Diagnosis** | | | | | |
| --- | --- | --- | --- | --- | --- |
|  |  | **Major depressive disorder** | | **Healthy controls** | |
| **Feature element** | **Uncorrected p-value** | **Mean** | **Standard deviation** | **Mean** | **Standard deviation** |
| FA: right medial orbitofrontal gyrus -  left rostral middle frontal gyrus | 1.61 • 10^-4^ | 3.41 • 10^-1^ | 2.35 • 10^-1^ | 4.89 • 10^-1^ | 1.35 • 10^-1^ |
| sSW: left pericalcarine cortex - left cuneus | 2.34 • 10^-4^ | 3.00• 10^1^ | 8.85• 10^0^ | 2.21• 10^1^ | 6.92• 10^0^ |
| FA: right precentral gyrus - left rostral anterior cingulate gyrus | 2.68 • 10^-4^ | 2.62 • 10^-1^ | 2.52 • 10^-1^ | 4.74 • 10^-1^ | 1.33 • 10^-1^ |
| AD: right precentral gyrus - left rostral anterior cingulate gyrus | 2.82 • 10^-4^ | 4.87 • 10^-4^ | 4.67 • 10^-4^ | 9.07 • 10^-4^ | 2.69 • 10^-4^ |
| RD: right amygdala - right pallidum | 4.00 • 10^-4^ | 3.06 • 10^-4^ | 7.69 • 10^-5^ | 3.69 • 10^-4^ | 5.70 • 10^-5^ |
| sSW: right medial orbitofrontal gyrus - right caudal anterior cingulate gyrus | 7.91 • 10^-4^ | 3.96 • 10^-1^ | 2.69 • 10^-1^ | 7.87 • 10^-1^ | 5.65 • 10^-1^ |
| CT: right posterior cingulate gyrus | 8.56 • 10^-4^ | 2.28• 10^0^ | 1.44 • 10^-1^ | 2.39• 10^0^ | 8.88 • 10^-2^ |
| sSW: right pallidum -  left banks of superior temporal sulcus | 8.76 • 10^-4^ | 7.12 • 10^-3^ | 6.64 • 10^-3^ | 2.82 • 10^-3^ | 3.58 • 10^-3^ |
| FA: right pars orbitalis -  right lateral orbitofrontal gyrus | 9.77 • 10^-4^ | 1.90 • 10^-1^ | 1.45 • 10^-1^ | 2.85 • 10^-1^ | 1.16 • 10^-1^ |
| FA: right rostral anterior cingulate gyrus - left rostral middle frontal gyrus | 1.04 • 10^-3^ | 3.43 • 10^-1^ | 2.21 • 10^-1^ | 4.85 • 10^-1^ | 9.92 • 10^-2^ |
| **Highest significant differences – Outcome prediction** | | | | | |
|  |  | **Negative outcome** | | **Positive outcome** | |
| **Feature element** | **Uncorrected p-value** | **Mean** | **Standard deviation** | **Mean** | **Standard deviation** |
| sSW: right precuneus - left fusiform gyrus | 1.43 • 10^-4^ | 6.88 • 10^-2^ | 4.88 • 10^-2^ | 1.70 • 10^-1^ | 8.02 • 10^-2^ |
| sSW: right hippocampus - left insula | 3.32 • 10^-4^ | 5.66 • 10^-3^ | 6.16 • 10^-3^ | 2.00 • 10^-2^ | 1.39 • 10^-2^ |
| MD: right caudal anterior cingulate gyrus - left rostral anterior cingulate gyrus | 3.87 • 10^-4^ | 8.63 • 10^-4^ | 2.57 • 10^-4^ | 5.74 • 10^-4^ | 1.85 • 10^-4^ |
| sSW: left paracentral gyrus - left inferior temporal gyrus | 4.83 • 10^-4^ | 2.13 • 10^-3^ | 1.35 • 10^-3^ | 7.61 • 10^-3^ | 5.89 • 10^-3^ |
| sFC: primary visual network - sensorimotor network | 7.08 • 10^-4^ | -8.23 • 10^-3^ | 2.17 • 10^-1^ | 2.14 • 10^-1^ | 1.34 • 10^-1^ |
| AD: right banks of superior temporal sulcus - right pallidum | 8.20 • 10^-4^ | 8.94 • 10^-4^ | 4.44 • 10^-5^ | 7.71 • 10^-4^ | 2.44 • 10^-4^ |
| RD: right medial orbitofrontal gyrus - right pallidum | 8.20 • 10^-4^ | 5.82 • 10^-4^ | 1.74 • 10^-4^ | 3.61 • 10^-4^ | 1.67 • 10^-4^ |
| RD: right cerebellum - right medial orbitofrontal gyrus | 9.44 • 10^-4^ | 4.83 • 10^-4^ | 4.72 • 10^-5^ | 3.11 • 10^-4^ | 1.98 • 10^-4^ |
| sSW: right precuneus - left caudal anterior cingulate gyrus | 9.49 • 10^-4^ | 6.99 • 10^-3^ | 7.56 • 10^-3^ | 2.21 • 10^-2^ | 1.42 • 10^-2^ |
| sSW: right caudal middle frontal gyrus - left isthmus cingulate gyrus | 9.86 • 10^-4^ | 2.12 • 10^-3^ | 1.57 • 10^-3^ | 4.87 • 10^-3^ | 2.63 • 10-3 |

**Supplementary Table S10** Top 10 most significant group differences between major depressive disorder and healthy controls (diagnosis) and positive and negative outcome ( outcome prediction). Abbreviations: CT = cortical thickness; sFC = static functional connectivity; sSW = sum of streamline weights; MD = mean diffusivity; FA = fractional anisotropy; AD = axial diffusivity; RD = radial diffusivity.

# References

Behzadi, Y., Restom, K., Liau, J., Liu, T.T., 2007. A Component Based Noise Correction Method (CompCor) for BOLD and Perfusion Based fMRI. NeuroImage 37, 90–101. https://doi.org/10.1016/j.neuroimage.2007.04.042

Bell, A.J., Sejnowski, T.J., 1995. An information-maximization approach to blind separation and blind deconvolution. Neural Comput. 7, 1129–1159. https://doi.org/10.1162/neco.1995.7.6.1129

Bernas, A., Aldenkamp, A.P., Zinger, S., 2018. Wavelet coherence-based classifier: A resting-state functional MRI study on neurodynamics in adolescents with high-functioning autism. Comput. Methods Programs Biomed. 154, 143–151. https://doi.org/10.1016/j.cmpb.2017.11.017

Bharti, K., Graham, S.J., Benatar, M., Briemberg, H., Chenji, S., Dupré, N., Dionne, A., Frayne, R., Genge, A., Korngut, L., Luk, C., Zinman, L., Kalra, S., Consortium (CALSNIC), for the C.A.N., 2022. Functional alterations in large-scale resting-state networks of amyotrophic lateral sclerosis: A multi-site study across Canada and the United States. PLOS ONE 17, e0269154. https://doi.org/10.1371/journal.pone.0269154

Calhoun, V.D., Adali, T., Pearlson, G.D., Pekar, J.J., 2001. A method for making group inferences from functional MRI data using independent component analysis. Hum. Brain Mapp. 14, 140–151. https://doi.org/10.1002/hbm.1048

Cîrstian, R., Pilmeyer, J., Bernas, A., Jansen, J.F.A., Breeuwer, M., Aldenkamp, A.P., Zinger, S., 2023. Objective biomarkers of depression: A study of Granger causality and wavelet coherence in resting-state fMRI. J. Neuroimaging 33, 404–414. https://doi.org/10.1111/jon.13085

Desikan, R.S., Ségonne, F., Fischl, B., Quinn, B.T., Dickerson, B.C., Blacker, D., Buckner, R.L., Dale, A.M., Maguire, R.P., Hyman, B.T., Albert, M.S., Killiany, R.J., 2006. An automated labeling system for subdividing the human cerebral cortex on MRI scans into gyral based regions of interest. NeuroImage 31, 968–980. https://doi.org/10.1016/j.neuroimage.2006.01.021

Dhollander, T., Mito, R., Raffelt, D., Connelly, A., 2019. Improved white matter response function estimation for 3-tissue constrained spherical deconvolution. Presented at the 27th International Society of Magnetic Resonance in Medicine, Montréal, Quebec, Canada.

DuPre, E., Salo, T., Markello, R., Kundu, P., Whitaker, K., Handwerker, D., 2021. TE-dependent analysis of multi-echo fMRI with tedana. J. Open Source Softw. 6, 3669. https://doi.org/10.21105/JOSS.03669

Fischl, B., Salat, D.H., Busa, E., Albert, M., Dieterich, M., Haselgrove, C., van der Kouwe, A., Killiany, R., Kennedy, D., Klaveness, S., Montillo, A., Makris, N., Rosen, B., Dale, A.M., 2002. Whole Brain Segmentation: Automated Labeling of Neuroanatomical Structures in the Human Brain. Neuron 33, 341–355. https://doi.org/10.1016/S0896-6273(02)00569-X

Glover, G.H., Li, T.-Q., Ress, D., 2000. Image-based method for retrospective correction of physiological motion effects in fMRI: RETROICOR. Magn. Reson. Med. 44, 162–167. https://doi.org/10.1002/1522-2594(200007)44:1<162::AID-MRM23>3.0.CO;2-E

Hariri, A.R., Bookheimer, S.Y., Mazziotta, J.C., 2000. Modulating emotional responses: effects of a neocortical network on the limbic system. Neuroreport 11, 43–48. https://doi.org/10.1097/00001756-200001170-00009

Iglesias, J.E., Augustinack, J.C., Nguyen, K., Player, C.M., Player, A., Wright, M., Roy, N., Frosch, M.P., McKee, A.C., Wald, L.L., Fischl, B., Van Leemput, K., 2015. A computational atlas of the hippocampal formation using ex vivo, ultra-high resolution MRI: Application to adaptive segmentation of in vivo MRI. NeuroImage 115, 117–137. https://doi.org/10.1016/j.neuroimage.2015.04.042

Iglesias, J.E., Van Leemput, K., Augustinack, J., Insausti, R., Fischl, B., Reuter, M., Alzheimer’s Disease Neuroimaging Initiative, 2016. Bayesian longitudinal segmentation of hippocampal substructures in brain MRI using subject-specific atlases. NeuroImage 141, 542–555. https://doi.org/10.1016/j.neuroimage.2016.07.020

Jenkinson, M., Beckmann, C.F., Behrens, T.E., Woolrich, M.W., Smith, S.M., 2012. FSL. NeuroImage 62, 782–790. https://doi.org/10.1016/j.neuroimage.2011.09.015.

Jenkinson, M., Smith, S., 2001. A global optimisation method for robust affine registration of brain images. Med. Image Anal. 5, 143–156. https://doi.org/10.1016/S1361-8415(01)00036-6

Jeurissen, B., Tournier, J.-D., Dhollander, T., Connelly, A., Sijbers, J., 2014. Multi-tissue constrained spherical deconvolution for improved analysis of multi-shell diffusion MRI data. NeuroImage 103, 411–426. https://doi.org/10.1016/j.neuroimage.2014.07.061

Kundu, P., Voon, V., Balchandani, P., Lombardo, M.V., Poser, B.A., Bandettini, P.A., 2017. Multi-echo fMRI: A review of applications in fMRI denoising and analysis of BOLD signals. NeuroImage, Cleaning up the fMRI time series: Mitigating noise with advanced acquisition and correction strategies 154, 59–80. https://doi.org/10.1016/j.neuroimage.2017.03.033

Merenstein, J.L., Zhao, J., Mullin, H.A., Rudolph, M.D., Song, A.W., Madden, D.J., 2023. High-resolution multi-shot diffusion imaging of structural networks in healthy neurocognitive aging. NeuroImage 275, 120191. https://doi.org/10.1016/j.neuroimage.2023.120191

Mueller, S., Costa, A., Keeser, D., Pogarell, O., Berman, A., Coates, U., Reiser, M.F., Riedel, M., Möller, H., Ettinger, U., Meindl, T., 2014. The effects of methylphenidate on whole brain intrinsic functional connectivity. Hum. Brain Mapp. 35, 5379–5388. https://doi.org/10.1002/hbm.22557

Pilmeyer, J., Hadjigeorgiou, G., Lamerichs, R.M.J.N., Breeuwer, M., Aldenkamp, A.P., Zinger, S., 2023. Spatial and Temporal Quality of Brain Networks for Different Multi-Echo fMRI Combination Methods. IEEE Access 11, 114536–114549. https://doi.org/10.1109/ACCESS.2023.3324183

Pilmeyer, J., Lamerichs, R., Ramsaransing, F., Jansen, J.F.A., Breeuwer, M., Zinger, S., 2024. Improved clinical outcome prediction in depression using neurodynamics in an emotional face-matching functional MRI task. Front. Psychiatry 15. https://doi.org/10.3389/fpsyt.2024.1255370

Posse, S., Wiese, S., Gembris, D., Mathiak, K., Kessler, C., Grosse‐Ruyken, M.-L., Elghahwagi, B., Richards, T., Dager, S.R., Kiselev, V.G., 1999. Enhancement of BOLD-contrast sensitivity by single-shot multi-echo functional MR imaging. Magn. Reson. Med. 42, 87–97. https://doi.org/10.1002/(SICI)1522-2594(199907)42:1<87::AID-MRM13>3.0.CO;2-O

Saygin, Z.M., Kliemann, D., Iglesias, J.E., van der Kouwe, A.J.W., Boyd, E., Reuter, M., Stevens, A., Van Leemput, K., McKee, A., Frosch, M.P., Fischl, B., Augustinack, J.C., Alzheimer’s Disease Neuroimaging Initiative, 2017. High-resolution magnetic resonance imaging reveals nuclei of the human amygdala: manual segmentation to automatic atlas. NeuroImage 155, 370–382. https://doi.org/10.1016/j.neuroimage.2017.04.046

Shi, Q., Chen, H., Jia, Q., Yuan, Z., Wang, J., Li, Y., Han, Z., Mo, D., Zhang, Y., 2020. Altered Granger Causal Connectivity of Resting-State Neural Networks in Patients With Leukoaraiosis-Associated Cognitive Impairment—A Cross-Sectional Study. Front. Neurol. 11.

Smith, R.E., Raffelt, D., Tournier, J.-D., Connelly, A., 2022. Quantitative streamlines tractography: methods and inter-subject normalisation. Aperture Neuro 1–25. https://doi.org/10.52294/ApertureNeuro.2022.2.NEOD9565

Smith, R.E., Tournier, J.-D., Calamante, F., Connelly, A., 2015. SIFT2: Enabling dense quantitative assessment of brain white matter connectivity using streamlines tractography. NeuroImage 119, 338–351. https://doi.org/10.1016/j.neuroimage.2015.06.092

Smith, R.E., Tournier, J.-D., Calamante, F., Connelly, A., 2012. Anatomically-constrained tractography: Improved diffusion MRI streamlines tractography through effective use of anatomical information. NeuroImage 62, 1924–1938. https://doi.org/10.1016/j.neuroimage.2012.06.005

Smith, S.M., Fox, P.T., Miller, K.L., Glahn, D.C., Fox, P.M., Mackay, C.E., Filippini, N., Watkins, K.E., Toro, R., Laird, A.R., Beckmann, C.F., 2009. Correspondence of the brain’s functional architecture during activation and rest. Proc. Natl. Acad. Sci. 106, 13040–13045. https://doi.org/10.1073/pnas.0905267106

Sorg, C., Manoliu, A., Neufang, S., Myers, N., Peters, H., Schwerthöffer, D., Scherr, M., Mühlau, M., Zimmer, C., Drzezga, A., Förstl, H., Bäuml, J., Eichele, T., Wohlschläger, A.M., Riedl, V., 2013. Increased Intrinsic Brain Activity in the Striatum Reflects Symptom Dimensions in Schizophrenia. Schizophr. Bull. 39, 387–395. https://doi.org/10.1093/schbul/sbr184

Tournier, J.-D., Smith, R., Raffelt, D., Tabbara, R., Dhollander, T., Pietsch, M., Christiaens, D., Jeurissen, B., Yeh, C.-H., Connelly, A., 2019. MRtrix3: A fast, flexible and open software framework for medical image processing and visualisation. NeuroImage 202, 116137. https://doi.org/10.1016/j.neuroimage.2019.116137

Townsend, J.D., Eberhart, N.K., Bookheimer, S.Y., Eisenberger, N.I., Foland-Ross, L.C., Cook, I.A., Sugar, C.A., Altshuler, L.L., 2010. fMRI activation in the amygdala and the orbitofrontal cortex in unmedicated subjects with major depressive disorder. Psychiatry Res. 183, 209–217. https://doi.org/10.1016/j.pscychresns.2010.06.001

Wang, X., Foryt, P., Ochs, R., Chung, J.-H., Wu, Y., Parrish, T., Ragin, A.B., 2011. Abnormalities in Resting-State Functional Connectivity in Early Human Immunodeficiency Virus Infection. Brain Connect. 1, 207–217. https://doi.org/10.1089/brain.2011.0016

Whitfield-Gabrieli, S., Nieto-Castanon, A., 2012. Conn: A Functional Connectivity Toolbox for Correlated and Anticorrelated Brain Networks. Brain Connect. 2, 125–141. https://doi.org/10.1089/brain.2012.0073
